# Downregulation of adipose LPL by PAR2 contributes to the development of hypertriglyceridemia

**DOI:** 10.1172/jci.insight.173240

**Published:** 2024-07-08

**Authors:** Yiheng Huang, Liujun Chen, Lisha Li, Yadan Qi, Haibin Tong, Hong Wu, Jinjie Xu, Lin Leng, Sukhinder Cheema, Guang Sun, Zhengyuan Xia, John McGuire, Brian Rodrigues, Lawrence H. Young, Richard Bucala, Dake Qi

**Affiliations:** 1 College of Pharmacy, Rady Faculty of Health Sciences, University of Manitoba, Winnipeg, Manitoba, Canada.; 2 College of Life and Environment Sciences, Wenzhou University, Wenzhou, Zhejiang, China.; 3 Institute of Cardiovascular Disease, Henan University of Chinese Medicine, Zhengzhou, Henan, China.; 4 Beijing Anding Hospital, Capital Medical University, Beijing, China.; 5 Department of Internal Medicine, Yale University School of Medicine, New Haven, Connecticut, USA.; 6 Department of Biochemistry, Faculty of Science, and; 7 Faculty of Medicine, Memorial University, St. John’s, Newfoundland, Canada.; 8 Guangdong Medical University, Zhanjiang, Guangdong, China.; 9 Department of Medical Biophysics, Schulich School of Medicine & Dentistry, Western University, London, Ontario, Canada.; 10 Faculty of Pharmaceutical Sciences, University of British Columbia, Vancouver, British Columbia, Canada.; 11 Department of Cellular and Molecular Physiology, Yale University School of Medicine, New Haven, Connecticut, USA.

**Keywords:** Metabolism, Adipose tissue, Cytokines, Signal transduction

## Abstract

Lipoprotein lipase (LPL) hydrolyzes circulating triglycerides (TGs), releasing fatty acids (FA) and promoting lipid storage in white adipose tissue (WAT). However, the mechanisms regulating adipose LPL and its relationship with the development of hypertriglyceridemia are largely unknown. WAT from obese humans exhibited high PAR2 expression, which was inversely correlated with the *LPL* gene. Decreased LPL expression was also inversely correlated with elevated plasma TG levels, suggesting that adipose PAR2 might regulate hypertriglyceridemia by downregulating LPL. In mice, aging and high palmitic acid diet (PD) increased PAR2 expression in WAT, which was associated with a high level of macrophage migration inhibitory factor (MIF). MIF downregulated LPL expression and activity in adipocytes by binding with CXCR2/4 receptors and inhibiting Akt phosphorylation. In a MIF overexpression model, high-circulating MIF levels suppressed adipose LPL, and this suppression was associated with increased plasma TGs but not FA. Following PD feeding, adipose LPL expression and activity were significantly reduced, and this reduction was reversed in *Par2^–/–^* mice. Recombinant MIF infusion restored high plasma MIF levels in *Par2^–/–^* mice, and the levels decreased LPL and attenuated adipocyte lipid storage, leading to hypertriglyceridemia. These data collectively suggest that downregulation of adipose LPL by PAR2/MIF may contribute to the development of hypertriglyceridemia.

## Abstract 2

MIF-mediated downregulation of adipose lipoprotein lipase contributes to hypertriglyceridemia.

## Graphical abstract

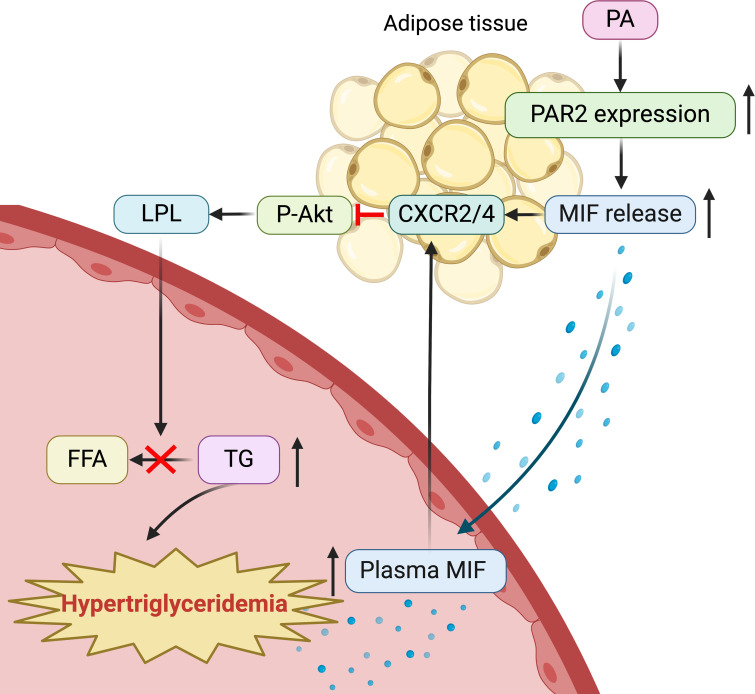

## Introduction

Lipoprotein lipase (LPL) plays a key role in breaking down plasma triglycerides (TGs) and promoting lipid storage in adipose tissue. LPL is ubiquitously expressed in white adipose tissue (WAT) including both s.c. and visceral adipose tissues (1). Transgenic defects in LPL in adipose tissue are associated with elevated plasma TG levels (2), suggesting a critical role for adipose LPL in regulating hypertriglyceridemia. LPL activity is attenuated in human s.c. adipose tissue associated with metabolic dysfunction (3), while weight loss results in increased LPL activity and expression (4). To date, the molecular mechanisms that regulate LPL in WAT and how they contribute to the regulation of hypertriglyceridemia remain unclear.

Macrophage migration inhibitory factor (MIF) is an evolutionarily conserved cytokine (5) that is highly expressed in visceral and s.c. adipose tissue. MIF is positively correlated with waist circumference or body fat percentage in patients with obesity (6, 7). Initially, MIF expression in WAT was thought to arise from infiltrating macrophages (8, 9). However, nonimmune cells such as adipose progenitors and adipocytes in WAT also release MIF under physiologic and pathologic conditions (10, 11). Circulating MIF levels are associated with metabolic dysfunction in the presence or absence of inflammation (9, 12), but its role in regulating hypertriglyceridemia is currently unknown.

Protease-activated receptors (PARs) belong to a unique class of GPCR expressed on various cell types, including endothelial cells (13) and adipocytes (12, 14). Following N-terminal cleavage, PARs activate intracellular G-protein signaling cascades (15). A cell-penetrating, lipidated PAR2 inhibitor, PZ-235 reduces fatty liver steatosis and hypertriglyceridemia by up to 50% (16), suggesting an inverse relationship between PAR2 and hypertriglyceridemia. Interestingly, PAR2 activation also induces *MIF* mRNA expression in human endothelial cells (17). Herein, in the present study, we investigated a hypothesis that PAR2 regulates hypertriglyceridemia through MIF. Our data indicate that increased PAR2 expression upregulates circulating MIF levels, which downregulate LPL expression in WAT. Alterations in LPL were associated with impaired clearance of plasma TG, which was associated with hypertriglyceridemia. Loss of PAR2 reduced plasma MIF levels, thereby abrogating the inhibitory effect of MIF on adipose LPL expression. Thus, loss of PAR2 increased plasma TG clearance and fat storage in WAT.

## Results

### Human obesity increases adipose PAR2 expression, which is associated with downregulation of LPL expression and hypertriglyceridemia.

**Figure 1:**
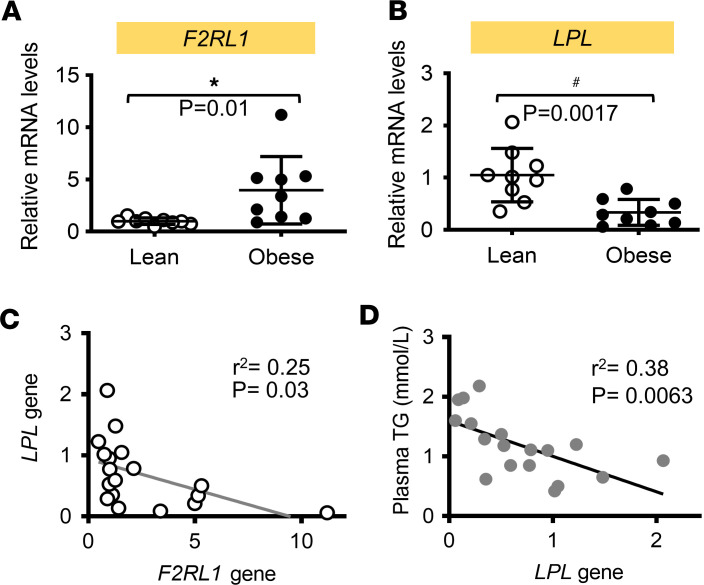
Human obesity increases adipose PAR2 expression, which is associated with downregulation of LPL expression and hypertriglyceridemia. (**A** and **B**) *F2RL1* and *LPL* mRNA expression in biopsied WAT from male patients including lean (age: 23.08 ± 2.15; BMI ≤ 25 kg/m^2^) and obese individuals (age: 24.6 ± 3.39; BMI > 30 kg/m^2^). (**C** and **D**) The correlation between *F2RL1* and *LPL* mRNA expression was shown in **C**, while the correlation between *LPL* mRNA expression and plasma TG levels was shown in **D**. *n* = 9 each group; all data are analyzed by Student’s *t* test (**A** and **B**) or Pearson correlation (**C** and **D**) and presented as mean ± SD. *P ≤ 0.05 increase vs. lean group in **A**; ^#^*P* ≤ 0.05 reduction vs. lean group in **B**.

We initially sought to establish the clinical relevance of PAR2 and LPL in WAT. Therefore, we sampled abdominal adipose tissue from lean (age: 23.08 ± 2.15; BMI < 25kg/m^2^) and obese (age: 24.6 ± 3.39; BMI > 30kg/m^2^) individuals with metabolic dysfunction (Supplemental Table 3; supplemental material available online with this article; https://doi.org/10.1172/jci.insight.173240DS1). We found that obesity was associated with increased expression of the PAR2 gene, *F2RL1* (Figure 1A), and decreased expression of the *LPL* gene (Figure 1B). *F2RL1* expression was inversely correlated with *LPL* gene expression (Figure 1C), which was inversely correlated with plasma TG levels (Figure 1D). These data suggest that *F2RL1* expression during obesity may repress adipose LPL, resulting in hypertriglyceridemia.

### Elevated PAR2 gene expression is associated with reduced LPL expression in WAT and hypertriglyceridemia following high palmitic acid diet feeding.

**Figure 2:**
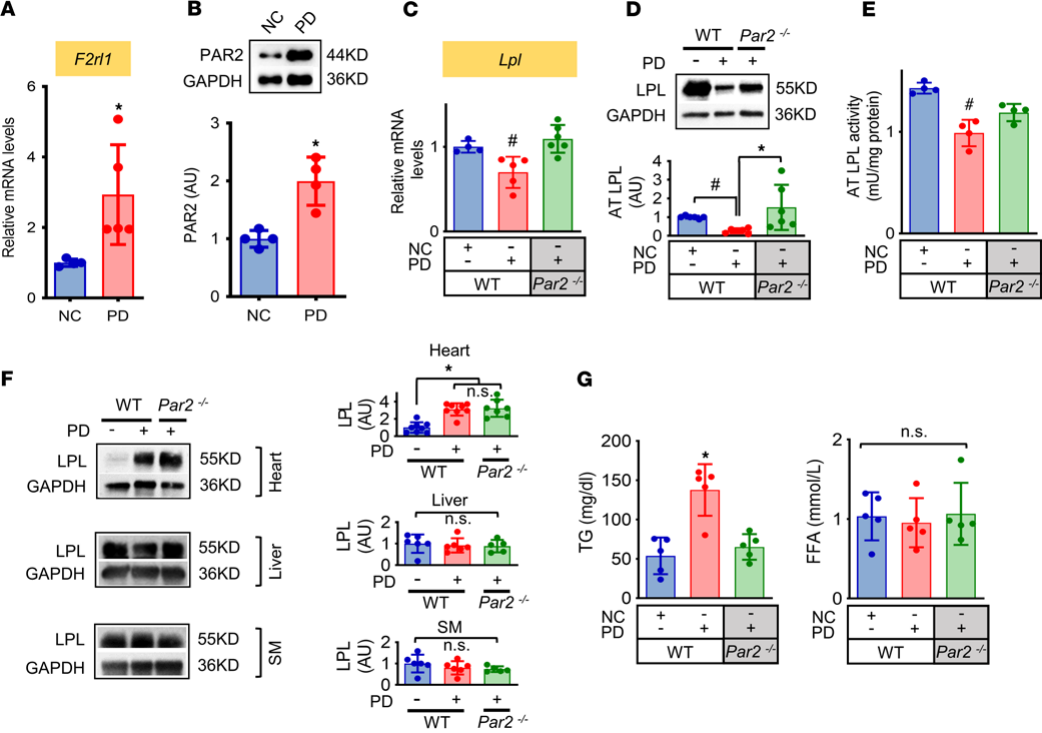
Elevated PAR2 gene expression is associated with reduced LPL expression in WAT and hypertriglyceridemia following high palmitic acid diet feeding. WT and *Par2^–/–^* mice (20-week-old) were fed with normal chow (NC) or a high palmitic acid diet (PD) for 8 weeks. (**A**–**E**) Measurement of *F2rl1* mRNA and PAR2 protein in adipose tissue by qPCR and Western blot (**A** and **B**) and quantification of adipose *LPL* gene and protein levels and activity (AT LPL) (**C**–**E**). (**F**) LPL protein levels in heart, liver, and skeletal muscle (SM) were evaluated by Western blot. (**G**) Plasma triglyceride (TG) and free fatty acid (FFA) levels of NC and PD mice. *n* = 4–7 each animal group. All data are analyzed by Student’s *t* test or 1-way ANOVA and presented as mean ± SD. *P ≤ 0.05 increase vs. NC in **A**, **B**, and **F** or vs. other groups in **G** or vs. WT PD in **D**; ^#^*P* ≤ 0.05 reduction vs. other groups in **C** and **E** or vs. WT NC.

We fed WT and *Par2^–/–^* mice (20 weeks) a high palmitic acid diet (PD) for 8 weeks. PD significantly upregulated PAR2 gene (*F2rl1*) and protein expression (Figure 2, A and B), with increased PAR2 downstream readout, ERK phosphorylation (Supplemental Figure 1A) in WAT. However, PAR2 expression and activation were not associated with any changes in circulating levels of tissue factor (Supplemental Figure 1B), suggesting the upregulation of PAR2 activation is probably not associated with tissue factor. Increased PAR2 expression correlated with decreased LPL expression and activity (Figure 2, C–E). In the absence of PAR2, PD was unable to reduce LPL (Figure 2, C–E). While PD significantly upregulated LPL expression in the heart, cardiac LPL was not affected by PAR2 deficiency (Figure 2F). In addition, LPL protein contents in the liver and skeletal muscle were unchanged following either high PD feeding or PAR2 KO (Figure 2F). These data collectively suggest that PAR2 expression specifically downregulates LPL in WAT rather than in the heart, liver and skeletal muscle.

Suppression of adipose LPL following PAR2 expression was associated with hypertriglyceridemia and was reversed in *Par2^–/–^* mice (Figure 2G). Interestingly, this regulation of plasma TGs occurred in the absence of changes in plasma free fatty acid (FFA) concentrations and liver and skeletal muscle lipid content (Figure 2G and Supplemental Figure 2, A and B). These results suggest that PAR2 expression in adipose tissue may reduce plasma TG clearance by modulating LPL following high-fat diet feeding in mice.

### PAR2 gene expression increases adipose MIF release and circulating MIF levels, thereby downregulating LPL expression and activation in WAT.

**Figure 3:**
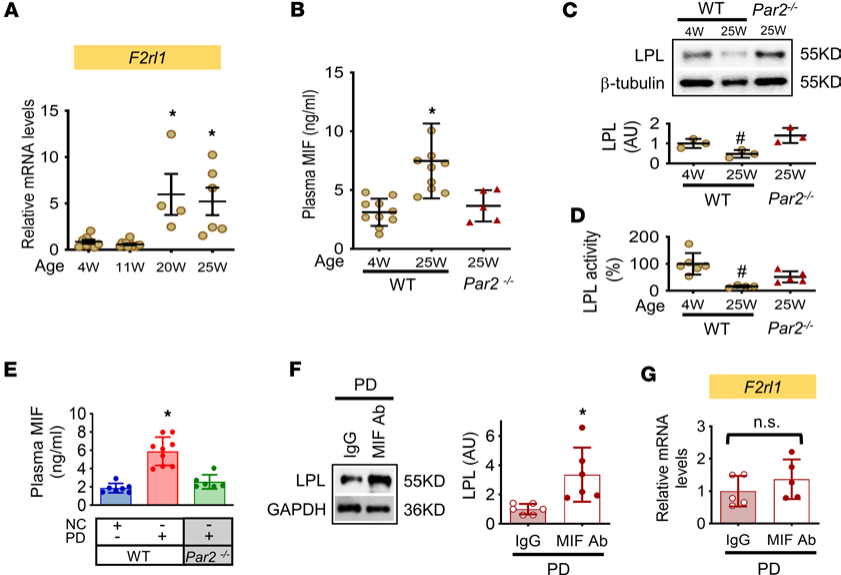
PAR2 gene expression increases adipose MIF release and circulating MIF levels, thereby downregulating LPL expression and activation in WAT. (**A**–**D**) Adipose tissues were collected from C57BL/6 WT mice at 4–25 weeks of age with normal chow feeding. *F2rl1* gene was measured by qPCR (**A**). Plasma MIF levels were evaluated in 4- and 25-week WT or *Par2^–/–^* mice by ELISA (**B**) and LPL protein expression and activity were measured in **C** and **D**. (**E**–**G**) WT and *Par2^–/–^* mice at 20 weeks were fed with normal chow (NC) or high palmitic acid diet (PD) feeding for 8 weeks. Plasma MIF levels were quantified subsequently in **E**. LPL expression (**F**) and *F2rl1* gene (**G**) were quantified in WT mice with high palmitic acid diet feeding following MIF neutralization with anti-MIF antibody (20 mg/kg, i.p. twice a week). *n* = 3–9 each animal group. All data are analyzed by Student’s *t* test or 1-way ANOVA and presented as mean ± SD. *P ≤ 0.05 increase vs. 4 weeks in **A**, vs. other groups in **B** and **E**, or vs. IgG in **F**; ^#^*P* ≤ 0.05 reduction vs. other groups in **C** and **D**.

We found that adipose *F2rl1* expression is age dependent. WAT from older mice (20–25 weeks) showed higher levels of *F2rl1* gene expression compared with 4-week-old mice (Figure 3A). Our recent study shows that PAR2 expression upregulates the release of MIF in adipose tissue (12). Accordingly, higher levels of PAR2 expression at 25 weeks were associated with increased plasma MIF levels (Figure 3B) and accompanied by decreased adipose MIF content but unchanged *Mif* gene expression (Supplemental Figure 3, A–C). High circulating MIF levels at 25 weeks were also associated with decreased LPL expression and activity compared with 4 weeks (Figure 3, C and D). Plasma MIF levels were further increased in 20-week WT mice fed a high PD (Figure 3E). In the absence of adipose PAR2, elevated plasma MIF levels were suppressed in aged mice or mice fed high PD (Figure 3, B and E), accompanied by reversed LPL expression and activities (Figure 2, C–E, and Figure 3, C and D). In high PD–fed WT mice, inhibition of LPL expression was abolished by neutralizing the MIF effect with anti-MIF antibody (Figure 3F). MIF neutralization did not affect *F2rl1* gene expression (Figure 3G). These results collectively suggest that MIF may mediate PAR2-downregulated LPL in WAT.

### MIF downregulates adipose LPL expression through a CXCR/Akt signaling pathway.

**Figure 4:**
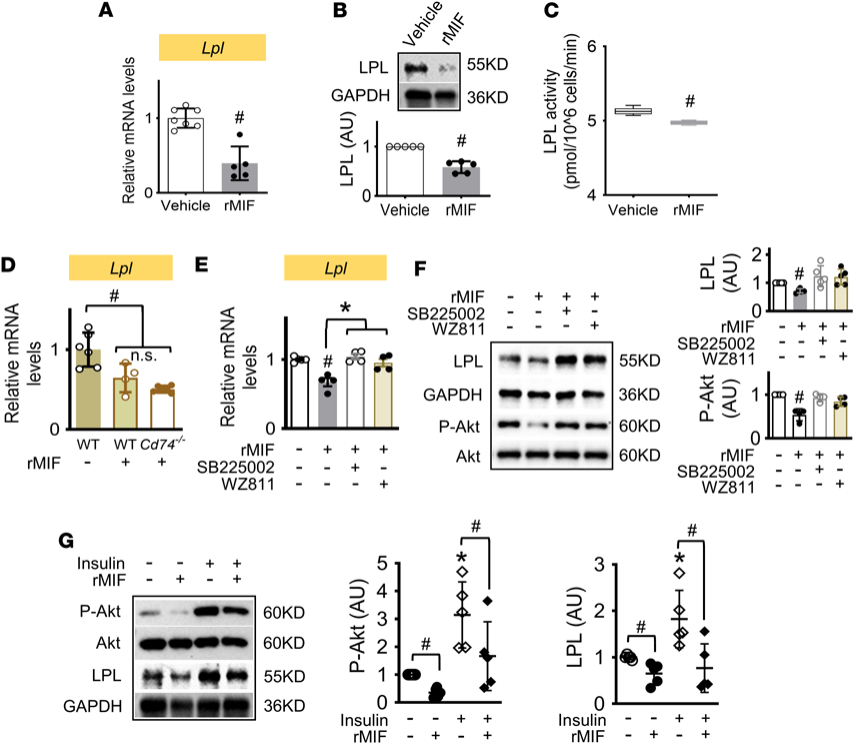
MIF downregulates adipose LPL expression through a CXCR/Akt signaling pathway. (**A**–**C**) Recombinant mouse MIF protein (400 ng/mL) was incubated with differentiated 3T3-L1 adipocytes for 24 hours, and LPL expression and activity were measured. Mature adipocytes were initially isolated from WT and *Cd74^–/–^* mice, and suspended cells were treated with vehicle or recombinant mouse MIF (rMIF, 400 ng/mL) for 24 hours. (**D**) *Lpl* gene was quantified by qPCR. In 3T3-L1 adipocytes, rMIF was incubated with the CXCR2 or CXCR4 inhibitors, SB225002 (400nM) and WZ811 (5μM). (**E** and **F**) The levels of *Lpl* gene and proteins and Akt phosphorylation were subsequently evaluated. (**G**) MIF regulated Akt phosphorylation and LPL protein expression in the presence of insulin were assessed by Western blot. All data are analyzed by Student’s *t* test or 1-way ANOVA and presented as mean ± SD. *P ≤ 0.05 increase vs. rMIF group in **E**, vs. other groups in **G**; ^#^*P* ≤ 0.05 reduction vs. vehicle in **A**–**D** and **G**, vs. other groups in **E** and **F**, vs. insulin in **G**.

We next investigated the direct effect of MIF on LPL expression in adipocytes. Recombinant mouse MIF protein (400 ng/mL) was incubated with 3T3-L1 differentiated adipocytes for 24 hours. LPL gene and protein expression and its activity were significantly suppressed (Figure 4, A–C). To further investigate the mechanism by which MIF downregulates LPL, we isolated adipocytes from WT and MIF receptor, CD74-KO (*Cd74^–/–^*) mice and subsequently treated with recombinant MIF for 24 hours. Interestingly, MIF-downregulated *Lpl* expression was not reversed by the deficiency of CD74 (Figure 4D). However, in 3T3-L1 adipocytes, the CXCR2 and -4 inhibitors, SB225002 and WZ811, respectively blocked MIF-induced downregulation of LPL expression (Figure 4, E and F). Taken together, these data suggest that MIF regulates LPL expression through binding with its noncognate CXCR2 and -4 receptors but not CD74 in adipocytes. Previous studies have shown that Akt upregulates *LPL* gene expression in human adipocytes as well as liver and mouse macrophages (18–20). Thus, we examined whether MIF regulates LPL expression through Akt. We found that MIF-induced downregulation of LPL expression was associated with a reduction in Akt phosphorylation that could be reversed by inhibition of CXCR2 and -4 (Figure 4F). Interestingly, LPL expression was significantly upregulated with activation of Akt following insulin treatment and was reversed by MIF (Figure 4G), suggesting that Akt may be an important mediator in modulating MIF-induced reduction of LPL expression in adipocytes.

### MIF overexpression induces high-circulating MIF levels, thereby suppressing adipose LPL and inducing hypertriglyceridemia.

**Figure 5:**
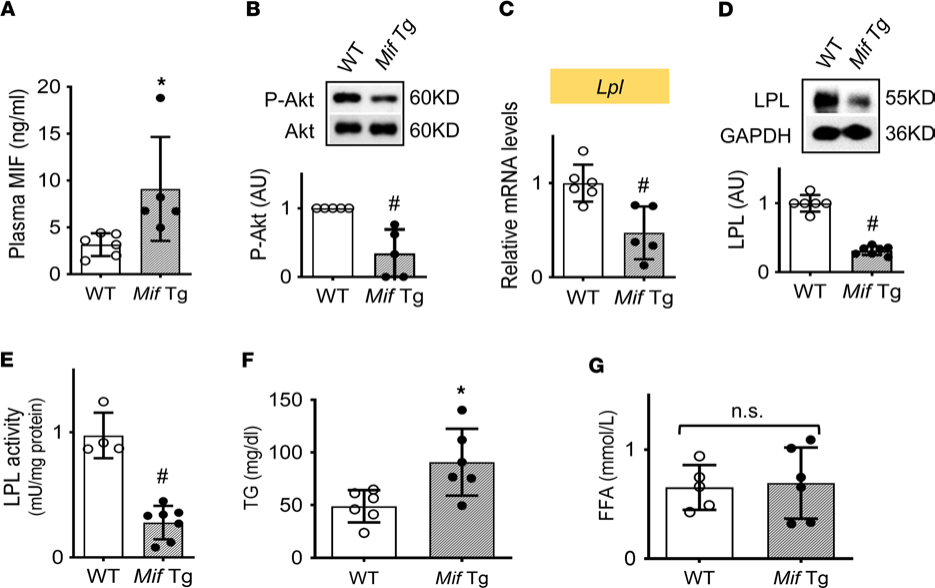
MIF overexpression induces high circulating MIF levels, thereby suppressing adipose LPL and inducing hypertriglyceridemia. (**A**) Plasma MIF levels were quantified by ELISA in WT and *Mif* lung Tg mice (25 weeks old). (**B**–**E**) Akt phosphorylation, *Lpl* mRNA and protein expression, and LPL activity were evaluated in their adipose tissues. (**F** and **G**) Plasma triglyceride (TG) and free fatty acid (FFA) levels in WT and TG mice. *n* = 4–6 each animal group. All data are analyzed by Student’s *t* test and presented as mean ± SD. *P ≤ 0.05 increase vs. WT in **A** and **F**; ^#^*P* ≤ 0.05 reduction vs. WT in **B**–**E**.

To further examine whether MIF downregulates LPL in WAT in vivo, we used a transgenic mouse model that overexpresses MIF in the lung (*Mif* lung Tg mice) (21). Plasma MIF levels were increased 2-fold in *Mif* lung Tg mice compared with WT littermates (Figure 5A). High-circulating plasma MIF concentrations were associated with inhibited Akt phosphorylation (Figure 5B) and reduced expression of *Lpl* gene and LPL protein in WAT (Figure 5, C and D), but not in liver and skeletal muscle (Supplemental Figure 4), suggesting that MIF specifically inhibits adipose LPL expression. Indeed, *Mif* lung Tg mice also had no detectable alterations in histological appearance and lipid storage of liver and skeletal muscle (Supplemental Figure 5). The reduction in adipose LPL expression was further reflected by attenuated LPL activity (Figure 5E). Downregulation of LPL in WAT was associated with increased plasma TG levels (Figure 5F), although plasma FFA levels were unchanged (Figure 5G). These findings overall suggest that MIF inhibition of LPL in WAT contributes to the development of hypertriglyceridemia.

### Restoration of high plasma MIF levels reverses high LPL in WAT of PAR2-deficient mice, resulting in hypertriglyceridemia.

**Figure 6:**
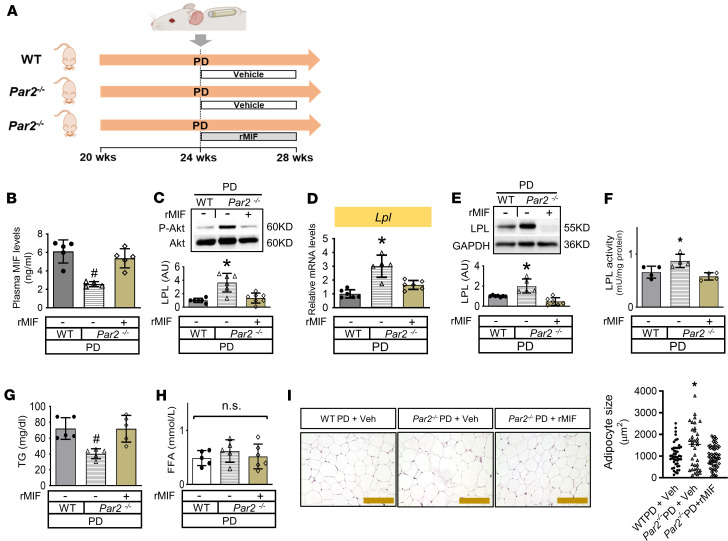
Restoration of high plasma MIF levels reverses high LPL in WAT of PAR2-deficient mice, resulting in hypertriglyceridemia. (**A**–**H**) Vehicle or recombinant mouse MIF (rMIF, 24 μg/day/kg) was administered i.v. by an osmotic pump to WT or *Par2* ^–/–^ mice half-way through the PD regiment (at the last 4 weeks) (**A**). Plasma MIF levels were quantified by ELISA in **B**. Adipose Akt phosphorylation (**C**); *Lpl* mRNA (**D**), protein (**E**), and activity (**F**); plasma TG (**G**); FFA (**H**); and H&E staining of adipose tissue (**I**) were also evaluated. Scale bars: 100 μM. *n* = 4–6 each animal group. All data are analyzed by 1-way ANOVA and presented as mean ± SD. *P ≤ 0.05 increase vs. other groups in **C**–**F** and **I**; ^#^*P* ≤ 0.05 reduction vs. other groups in **B** and **G**.

*Par2^–/–^* mice were fed a high PD and received vehicle or recombinant MIF via osmotic pump during the final 4-week feeding period (Figure 6A). MIF infusion reversed plasma MIF levels (Figure 6B). Akt phosphorylation (Figure 6C) and LPL expression and activity (Figure 6, D–F) in WAT was decreased after MIF infusion in *Par2^–/–^* mice. Alterations in LPL were associated with attenuated plasma TG clearance (Figure 6G) and reduced lipid storage in WAT (Figure 6I), although plasma FFA levels were unchanged (Figure 6H). Under these conditions, MIF infusion did not affect the expression of inflammatory factors *Tnfa*, *Il6*, and *Il1b* in WAT (Supplemental Figure 6). These data suggest that MIF plays a key role in the regulation of PAR2-mediated adipose LPL expression and the development of hypertriglyceridemia.

## Discussion

Obesity is inversely correlated with LPL in WAT. However, the molecular mechanisms mediating LPL during obesity are largely unknown. Our current study identifies a regulatory mechanism of LPL in WAT which contributes to hypertriglyceridemia. We found that the expression of PAR2 was significantly increased in WAT isolated from patients with obesity and was inversely correlated with the *LPL* gene. Reduced LPL expression was also negatively correlated with elevated plasma TG levels, suggesting that adipose PAR2 may contribute to the development of hyperlipidemia through downregulation of LPL. In animal models, adipose PAR2 expression was associated with high plasma MIF, a cytokine that downregulates LPL expression and activity through binding with CXCR2/4 and inhibiting Akt phosphorylation in adipocytes. Thus, following high-fat diet feeding, PAR2 deficiency attenuated the rise in plasma MIF levels, reversed LPL expression and activity in WAT, and thus corrected hypertriglyceridemia. These data together suggest that downregulation of adipose LPL by PAR2/MIF is an important mechanism for the development of hypertriglyceridemia.

Hypertriglyceridemia is an important biomarker of metabolic dysfunction in abdominal obesity (22). It also increases the risk of cardiovascular disease even in the presence of optimized LDL cholesterol levels (23). Hypertriglyceridemia is associated with the activation of LPL, which hydrolyzes plasma TGs into nonesterified fatty acids (24). Decreased LPL, for instance, leads to increased plasma TG concentrations in patients with type 2 diabetes (12). LPL is produced by many tissues, including WAT, skeletal muscle, and heart. Overexpression of LPL in skeletal muscle protects against excess weight gain by increasing TG accumulation in skeletal muscle (25). LPL deficiency in the heart results in hypertriglyceridemia and cardiac dysfunction (26). WAT is a key organ for lipid storage, so adipose LPL acts as a gatekeeper for directing TGs to WAT. Indeed, adipose LPL is impaired in obesity (27), and underexpression of adipose LPL reduces fatty acid entry into WAT (28). Our current study shows that manipulation of LPL in WAT but not in skeletal muscle, liver, or heart is associated with hypertriglyceridemia.

LPL can be regulated at transcriptional, posttranscriptional, and posttranslational levels. The present study identifies a transcriptional regulation of LPL by PAR2, a member of the GPCR family. PAR2 is expressed in various cell types, including endothelial cells (13) and adipocytes (12, 14). Our clinical data demonstrate that obesity upregulates PAR2 expression in WAT, which inversely correlates with *LPL* gene expression, leading to hypertriglyceridemia. A high-fat diet successfully mimics all genotypes and metabolic phenotypes in mouse models that can be abolished by PAR2 KO. Although a previous study indicates that PAR2 accelerates adipocyte differentiation (29), our recent data did not observe any difference in the expression of adipogenesis marker PPARγ between patients who are lean and patients with obesity or between WT and *Par2^–/–^* mice with high fat diet feeding (12). Thus, PAR2 expression may have a differentiation-independent effect on regulating LPL in adipose tissue. Furthermore, it should be noted that increased PAR2 expression may be related to cleavage and activation of PAR2 by proteases, such as plasmin. A recent study indeed demonstrated that angiopoietin-like protein (ANGPTL) 4/8 complex mediates plasmin generation, thereby upregulating LPL activity and postprandial TG hydrolysis (30). However, it is unclear whether plasmin is also involved in the regulation of *Lpl* gene expression by upregulating PAR2 activation and concomitantly increasing PAR2 expression.

Macrophage MIF is an evolutionarily conserved cytokine generally recognized as an upstream regulator of the innate immune response (5). Other work has highlighted the role of MIF in promoting metabolic dysfunction (31). However, whether MIF regulates LPL and hypertriglyceridemia was previously unknown. Our present data indicate that MIF directly represses the transcription of LPL in adipocytes. High plasma MIF concentrations in the MIF overexpression model reduced adipose but not liver or skeletal muscle LPL expression. Interestingly, while the inflammatory state may affect preheparin LPL mass in rheumatoid arthritis (32), MIF-regulated LPL transcription is independent of inflammation. Together, these data suggest a direct relationship between MIF and LPL expression in the absence of inflammation in adipose tissue.

LPL transcription was upregulated by Akt in human adipocytes and in liver and mouse macrophages (18–20). MIF downregulates Akt phosphorylation in adipocytes in the presence or absence of insulin. Our data further demonstrate that MIF inhibits *Lpl* gene expression by downregulating Akt, and this effect was associated with the classical chemokine receptors CXCR2/4, which are both noncognate receptors for MIF. Although CXCR2/4 and CD74 both have been identified as the MIF receptors in immune cells, CD74 is considered to be the primary receptor regulating MIF cell functions in nonimmune cells, including cardiomyocytes, hepatocytes, and renal cells (33–35). Our recent studies also have shown that MIF inhibits hormone-sensitive lipase and lipolysis by binding to CD74 in adipocytes (36). However, the inhibitory effect of MIF on Akt/LPL signaling in adipocytes depends on CXCR2/4 but not CD74. The downstream mechanisms by which MIF activates CXCR2/4 in adipocytes to inhibit LPL is currently unclear but is deserving of further investigation.

PAR2 activation induces *MIF* mRNA expression in human endothelial cells (17), suggesting a possible link between PAR2 activity and regulation of MIF expression. However, our recent study showed that the expression and activation of PAR2 promotes the release of MIF but not its expression in adipose tissue in the absence of inflammation (12). Thus, PAR2 activation may repress LPL gene and activity in adipose tissue by mediating adipose MIF release. Indeed, antibody neutralization of plasma MIF following high fat diet feeding demonstrated a reversal of LPL expression, while maintaining high levels of PAR2 expression, suggesting that a critical role for MIF in PAR2-mediated LPL expression in adipose tissue.

PAR2-KO mice with reduced plasma MIF levels are protected from hypertriglyceridemia, an effect associated with adipocyte hypertrophy, indicating increased fatty acid uptake and storage. Plasma TGs are absorbed by WAT, which reduces lipid accumulation and potential toxicity in the liver and skeletal muscle (37). Although we did not observe differences in lipid storage in liver or skeletal muscle, MIF-mediated changes may be important later in the disease, such as the development of steatosis.

In conclusion, we have identified a role for PAR2 in regulating hypertriglyceridemia. Blocking fatty acid–induced PAR2 expression and/or reducing MIF secretion from WAT would be an important strategy to restore adipose LPL expression. Optimization of LPL expression and activity in WAT will reduce plasma TG levels, which can ultimately reduce the incidence of cardiovascular disease in conjunction with successful LDL-C reduction (23). Further work is needed to investigate the therapeutic applicability of these strategies in clinical settings for patients with obesity or type 2 diabetes.

### Limitations of the study.

We found that PAR2 expression is associated with increased PAR2 activation in adipocytes and that FA-induced upregulation of PAR2 transcription likely increases PAR2 activation by augmenting the amount of cell-surface PAR2. Interestingly, our data show that circulating levels of tissue factor are not accompanied with alterations in adipose PAR2, suggesting that tissue factor may not be the protease to cleave and activate PAR2 in mouse models. Given the evidence that obesity is often associated with high levels of circulating proteases, such as thrombin and tryptase (38, 39), targeting specific proteases that may contribute to PAR2 activation will be of interest for our future study.

## Methods

### Sex as a biological variable.

Our current study only involved male mice because our human data were obtained from a previous overfeeding study in which adipose and blood samples were collected from male patients. However, we believe our results will be important for both men and women due to the prevalence of hypertriglyceridemia in both sexes.

### Patients.

Human mRNA samples were obtained from a previous overfeeding study (Guang Sun at Memorial University of Newfoundland, St. John’s, Newfoundland and Labrador, Canada) to investigate the effects of a positive energy balance on endocrine factors and glucose and lipid metabolism, which has been approved by Newfoundland and Labrador Health Research Ethics Board (HREB) (19). We also obtained an ethic approval for a secondary use of these samples for the current study (Research portal file no. 20200635). Plasma MIF level was determined with an ELISA method according to the protocol from R&D Systems. Human PCR data were analyzed using *t* test; all significant levels were 2-tailed tested, and a *P* value of less than 0.05 was considered as statistically significant.

### Experimental animals.

*Par2^–/–^* mice on C57BL/6 background were originally obtained from John McGuire’s laboratory and bred with WT mice (The Jackson Laboratory) to generate *Par2^–/–^* mice and WT littermates for the designed experiments. MIF lung transgenic (*Mif* Lung Tg) (21) and CD74-KO (*Cd74^–/–^*) mice were generated in house. All WT and transgenic mice were bred in the Health Science Center Animal Facility in Memorial University of Newfoundland or the Animal Care Centre of University of Manitoba, Canada. They were housed in individual IVC cages with an artificial 12:12-hour light/dark cycle at room temperature and fed with either normal chow or high palmitic oil diet (41% palmitic oil; 170100, Envigo Teklad Diets) for 8 weeks.

### Recombinant mouse MIF infusion and MIF neutralization.

WT and mouse *Par2^–/–^* mice at 20 weeks were initially fed with high PD for 4 weeks. Jugular vein was then cannulated and recombinant mouse MIF (24 μg/day/kg) or vehicle was injected via a miniosmotic pump implanted in a s.c. pocket (Alzet model 1004) into the mice accompanied with high PD feeding for the following 4 weeks. In a separate experiment, plasma MIF was neutralized with anti-MIF antibody (20 mg/kg, i.p. twice a week, in house) during high fat feeding.

### 3T3-L1 cell culture.

3T3-L1 adipocytes (American Type Culture Collection) were cultured and differentiated as described previously (12). Before all experiments, cells were briefly serum-starved in DMEM-0.5% FBS for 8 hours.

### Antibodies and reagents.

Antibodies against LPL (catalog ab21356), PAR2 (catalog ab180953), phosphor-Akt (Ser^473^) (catalog 9271S) and total Akt (catalog 9272S), MIF (catalog TP234), phosphor-ERK (catalog 4376S) and total ERK (catalog 4695S), β-tubulin (catalog MA5-16308), and GAPDH (catalog 2118S) were purchased from Abcam, Invitrogen, Cell Signaling, and Torrey Pines Biolabs, respectively. Recombinant mouse MIF was purified from a high yield *E*. *coli* expression system by fast protein liquid chromatography (FPLC) followed by C8 chromatography to remove endotoxin (40). Mouse plasma MIF concentrations were measured by a 1-step sandwich enzyme-linked immunosorbent assay from R&D Systems as previously described (12). Plasma levels of TG and FFA were measured by L-type TG M assay kit and nonesterified fatty acid (NEFA) assay kit from FUJIFILM Wako Diagnostics. LPL activity was detected by the LPL assay kit from Abcam. The CXCR2 inhibitor SB225002, CXCR4 inhibitor WZ811, and insulin were purchased from Sigma-Aldrich.

### Expression and phosphorylation analyses.

The transcript levels for human and mouse *F2rl1,* *Lpl*, and *Gapdh genes* (Supplemental Table 1 and 2) were measured by qPCR (41). PAR2, LPL, β-tubulin, GAPDH, and phosphorylation and total levels of Akt in adipose tissue or cells were evaluated by Western blot.

### Histology.

H&E staining was performed to identify adipocyte hypertrophy in adipose tissue as described previously (41).

### Statistics.

One-way ANOVA with Tukey’s post hoc tests or 2-tailed Student *t* test was used to determine differences between group mean values. The level of statistical significance was set at *P* < 0.05.

### Study approval.

The human study was approved by Newfoundland and Labrador HREB (research portal file no. 20200635) and all human experiments were conducted in accordance with the Declaration of Helsinki. All study-related procedures were carried out with written informed consent. All experiments involving mice were conducted in accordance with the *Guide for the Care and Use of Laboratory Animals* (National Academies Press, 2011) and were approved by the IACUC of Memorial University of Newfoundland and University of Manitoba.

### Data availability.

Values for all data points in graphs are reported in the Supporting Data Values file.

## Author contributions

YH performed the major experiments. LC, L Li, SC, JX, and YQ participated in animal studies. HT, HW, L Leng, ZX, BR, and RB contributed intellectually to data analysis and manuscript editing. JM contributed to preparing the animal models. GS provided key supports in human studies. LHY and RB provided overall scientific support for the research project, and DQ designed and managed the research. All authors read and approved the final manuscript. DQ is the guarantor of this work and, as such, had full access to all the data in the study and takes responsibility for the integrity of the data and the accuracy of the data analysis.

## Supplementary Material

Supplemental data

Unedited blot and gel images

Supporting data values

## Version 1 07/08/2024

Electronic publication
