# Supplementary material for: Downregulation of adipose LPL by PAR2 contributes to the development of hypertriglyceridemia
Source: JCI Insight. 2024 Jul 8;9(13):e173240. doi: 10.1172/jci.insight.173240 (PMC11383372; doi:10.1172/jci.insight.173240)
Supplement: Supplemental data [file jciinsight-9-173240-s047.docx]

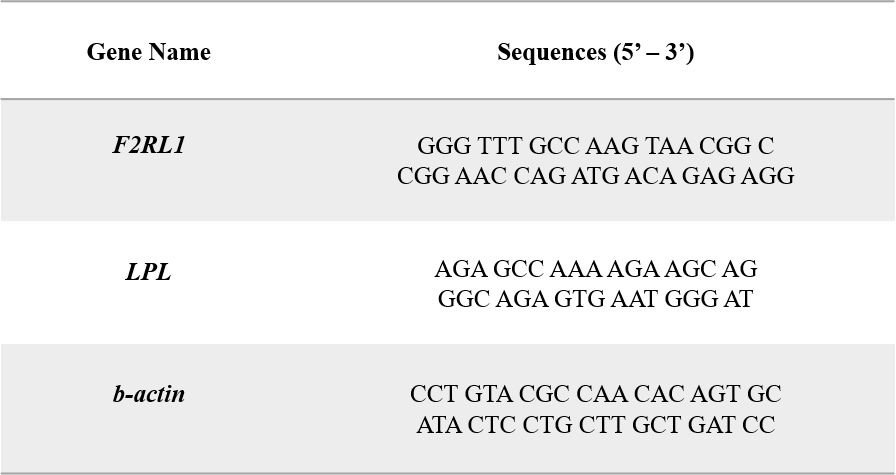


**Supplemental Table 1.** List of human PCR primer sequences.

**Supplemental Table 2.** List of mouse PCR primer sequences.

| Gene Name | Sequences (5’ – 3’) |
| --- | --- |
| *F2rl1* | AAC ATC ACC ACC TGT CAC GA  CAC GTA GGC AGA CGC AGT AA |
| *Mif* | CGG ACC GGG TCT ACA TAC A  TCA AGC GAA GGT GGA ACC GTT |
| *Lpl* | AAT TTG CTT TCG ATG TCT GAG AA  CAG AGT TTG ACC GCC TTC C |
| *Tnfa* | CAG GCG GTG CCT ATG TCT C  CGA TCA CCC CGA AGT TCA GTA G |
| *Il6* | GAG GAT ACC ACT CCC AAC AGA CC  AAG TGC ATC ATC GTT GTT CAT ACA |
| *Il1b* | TGG TGT GTG ACG TTC CCA TT  CAG CAC GAG GCT TTT TTG TTG |
| *Gapdh* | ATG TGT CCG TCG TGG ATC TGA  TGC CTG CTT CAC CAC CTT CTT |

|  | **Lean** | **Obese** | ***P*-value** |
| --- | --- | --- | --- |
| Age (years) | 23.08±2.15 | 24.60±3.39 | 0.2717 |
| BMI (kg/m^2^) | 21.50±1.62 | 33.01±3.54 | <0.0001 |
| Glucose (mmol/L) | 5.10±0.43 | 5.69±0.78 | 0.0670 |
| Insulin (pmol/L) | 32.35±17.07 | 123.01±80.01 | 0.0050 |
| TG (mmol/L) | 0.87±0.29 | 1.63±0.38 | 0.0009 |
| HDL (mmol/L) | 1.47±0.31 | 1.10±0.35 | 0.0173 |
| LDL (mmol/L) | 2.40±0.69 | 3.10±0.85 | 0.1243 |

**Supplemental Table 3.** General characteristics of human subjects

**Supplemental Figure 1** *High palmitic acid diet upregulates ERK phosphorylation but not affect circulating levels of tissue factor.* The WT and *Par2 ^-/-^* mice at 20 weeks were fed with normal chow (NC) or high palmitic acid diet (PD) for 8 weeks and the phosphorylation of ERK in adipose tissue (**A**) was evaluated by western blot. The levels of tissue factor were quantified in plasma by ELISA kit (**B**). n=4-8 for each group. Mean ± SD in all the panels. 2-tailed Student’s *t* test was performed for statistical analysis. The n.s. represents no significance.

**Supplemental Figure 2** *High palmitic acid diet feeding for 8 weeks did not affect lipid storage in liver and skeletal muscle in WT and Par2 ^-/-^ mice.* The WT and *Par2 ^-/-^* mice at 20 weeks were fed with normal chow (NC) or high palmitic acid diet (PD) for 8 weeks and the lipid storage in skeletal muscle and liver was quantified by oil red O staining (**A**) or enzymatic methods (**B**). n=3-5 for each group. Mean ± SD in all the panels; One-way ANOVA was performed for statistical analysis in (**B**). The n.s. represents no significance.

**Supplemental Figure 3** *Alterations of* *MIF contents in adipose tissue with age.* The WT mice from 4 to 25 weeks were euthanized and the MIF gene and protein levels in adipose tissue were quantified by Western blot (**A**), immunohistochemistry (**B**), or qPCR (**C**) (n=4-8 each group). Mean ± SD in all the panels; One-way ANOVA and 2-tailed Student’s *t* test were performed for statistical analysis. #P<0.05 reduction vs. 4 weeks. The n.s. represents no significance.

**Supplemental Figure 4** *Mif lung Tg mice have unchanged LPL protein levels in liver and skeletal muscle.* The WT (littermates) and *Mif* lung Tg mice at 25 weeks were euthanized and the LPL protein levels in liver (**A**) and skeletal muscle (**B**) were quantified by Western blot (n=3 each group). Mean ± SD in all the panels; 2-tailed Student’s *t* test was performed for statistical analysis. The n.s. represents no significance.

**Supplemental Figure 5** *The histological characteristics of WT and Mif lung Tg mice.* Adipose tissue, skeletal muscle and liver were isolated from WT and *Mif* lung Tg mice at 25 weeks for HE (**A**) and oil red O staining (**B**).

**Supplemental Figure 6** *The gene expression of inflammatory factors in adipose tissue from WT and Par2 ^-/-^mice following high palmitic oil diet feeding with or without MIF infusion.* The WT and *Par2 ^-/-^* mice at 20 weeks were fed with normal chow or high palmitic oil diet (PD) for 4 weeks. Recombinant mouse MIF (rMIF, 24 g/day/kg) were given by osmotic pump with PD for 4 weeks. The gene expression of *TNF-α* (**A**), *IL-1β* (**B**) and *IL-6* (**C**) in adipose tissue was quantified by qPCR. N=5 for each group. All data are analyzed by 1-way ANOVA and presented as mean ± SD.
