# Supplementary material for: Downregulation of adipose LPL by PAR2 contributes to the development of hypertriglyceridemia
Source: JCI Insight. 2024 Jul 8;9(13):e173240. doi: 10.1172/jci.insight.173240 (PMC11383372; doi:10.1172/jci.insight.173240)

Figure 2B

The PVDF membrane was initially stained with PAR2 antibody

The same membrane was subsequently stained with GAPDH antibody and exposed again

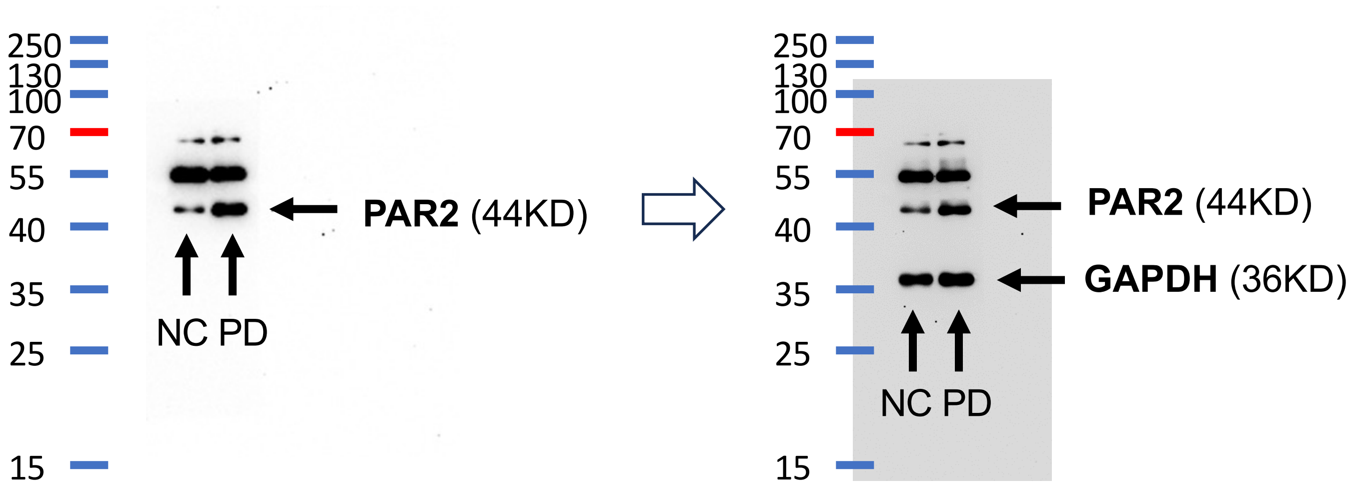

Figure 2D

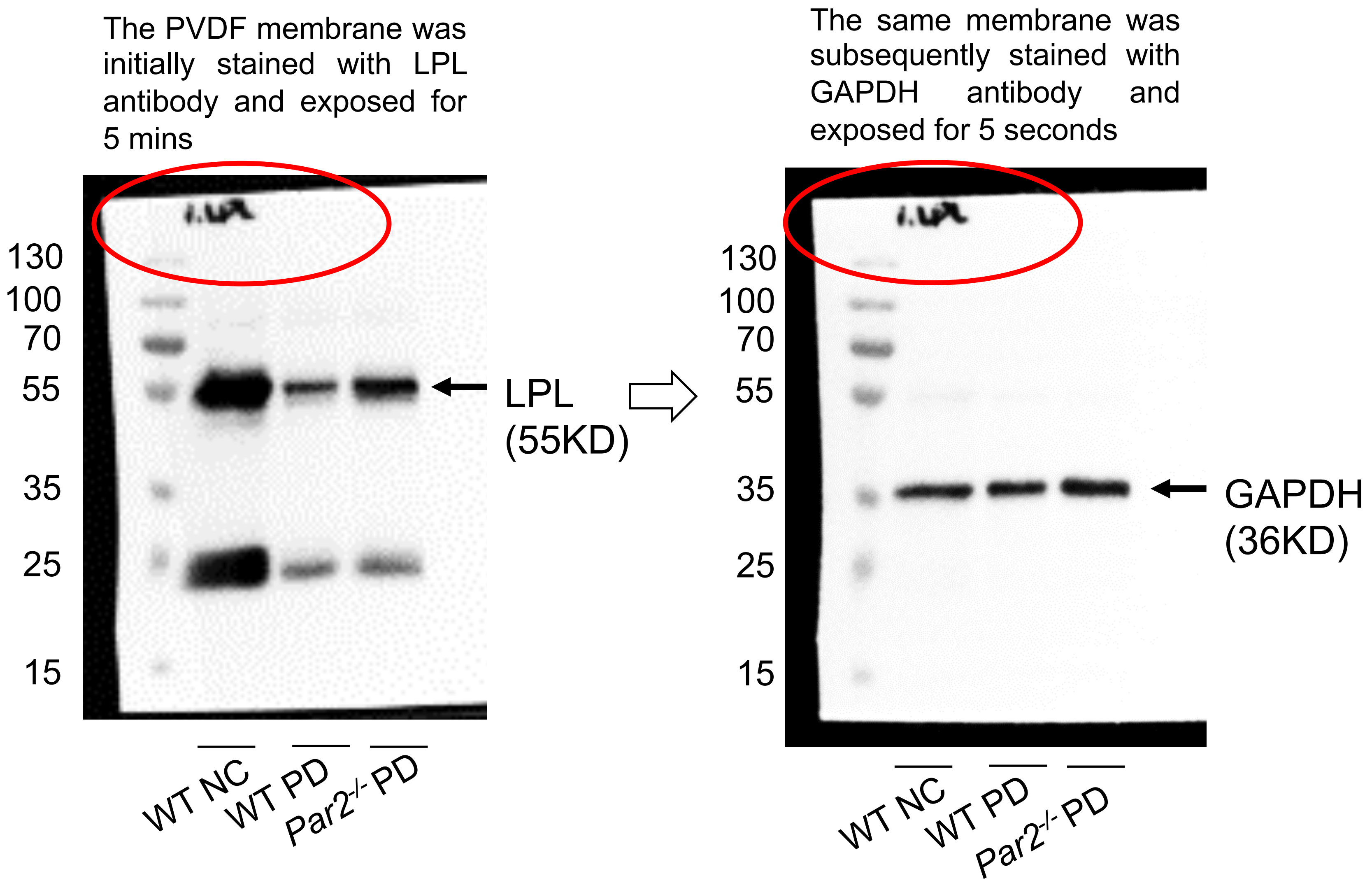
The PVDF membrane was initially stained with LPL antibody and exposed for 5 mins
130
100
70
55
35
25
15
LPL (55KD)
WT NC
WT PD
Par2-/- PD
The same membrane was subsequently stained with GAPDH antibody and exposed for 5 seconds
130
100
70
55
35
25
15
GAPDH (36KD)
WT NC
WT PD
Par2-/- PD

Figure 2F Heart

The PVDF membrane was initially stained with LPL antibody and exposed for 5 mins

The same membrane was subsequently stained with GAPDH antibody and exposed for 5 seconds

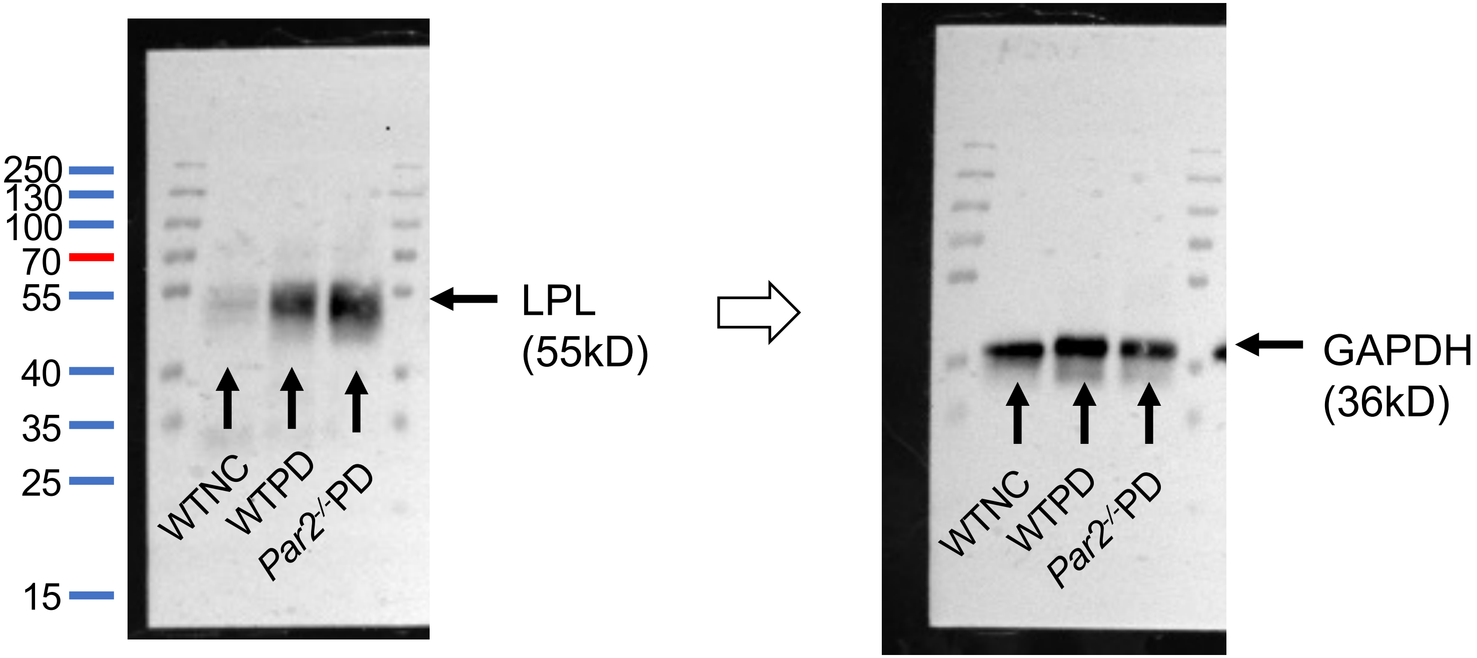

Figure 2F Liver

The PVDF membrane was initially stained with LPL antibody and exposed for 5 mins

The same membrane was subsequently stained with GAPDH antibody and exposed for 5 seconds

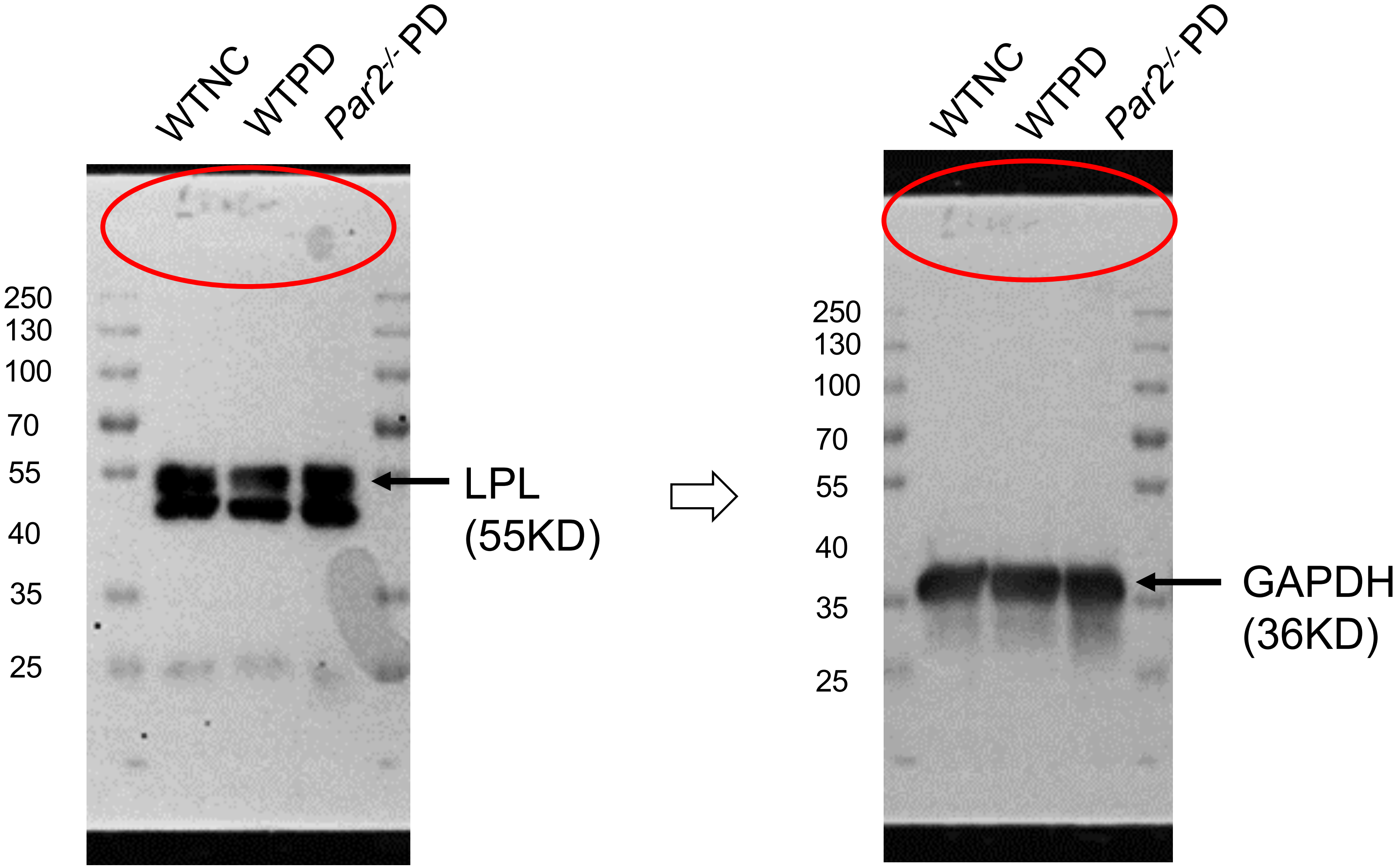

Figure 2F SM

The PVDF membrane was initially stained with LPL antibody and exposed for 5 mins

The same membrane was subsequently stained with GAPDH antibody and exposed for 5 seconds

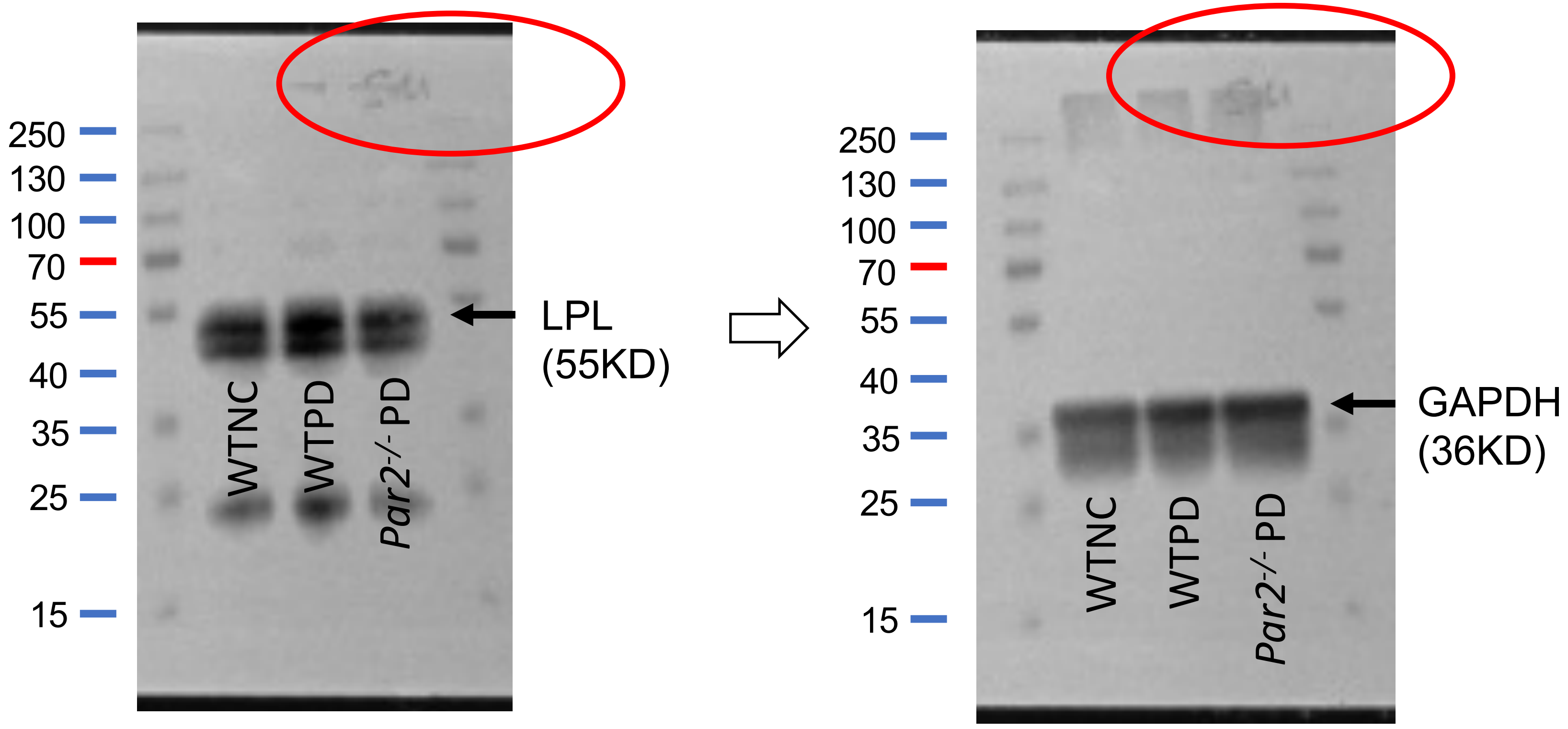

Figure 3C

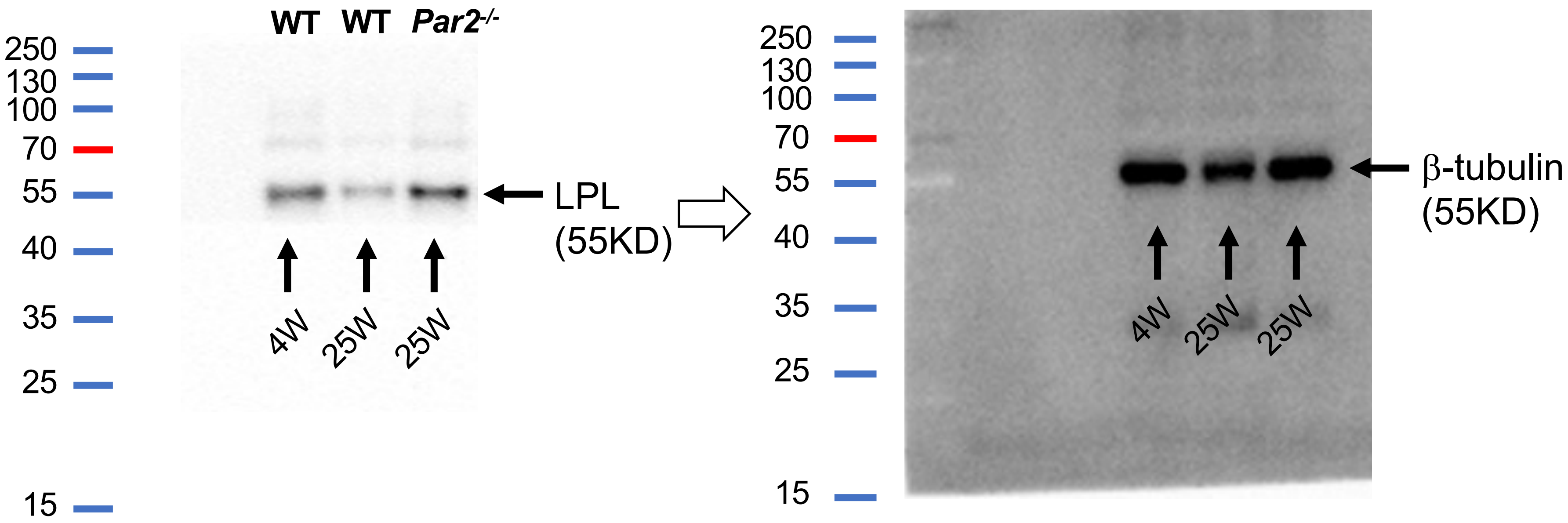
WT
WT
Par2-/-
250
130
100
70
55
40
35
25
15
LPL
(55KD)
4W
25W
25W
β-tubulin
(55KD)

Figure 3F

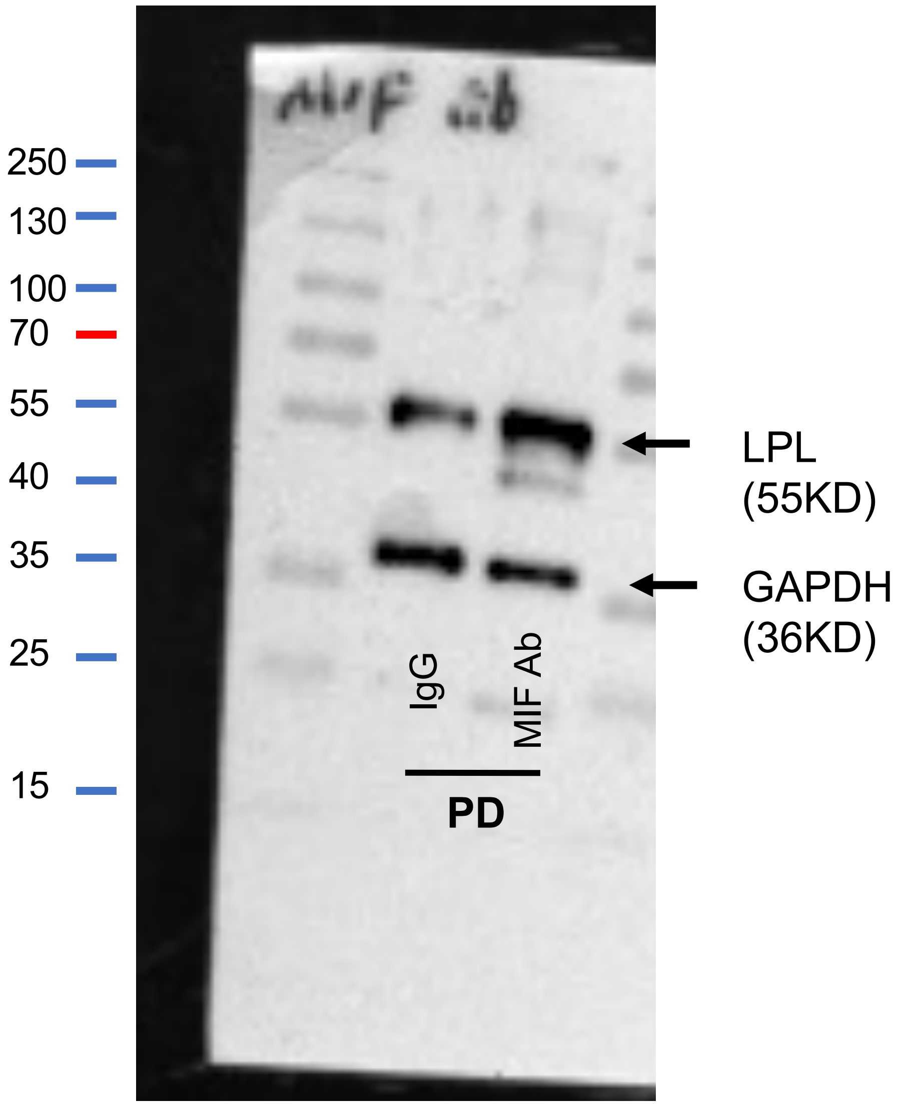

Figure 4B

The PVDF membrane was initially stained with LPL antibody and exposed for 5 mins

The same membrane was subsequently stained with GAPDH antibody and exposed for 5 seconds

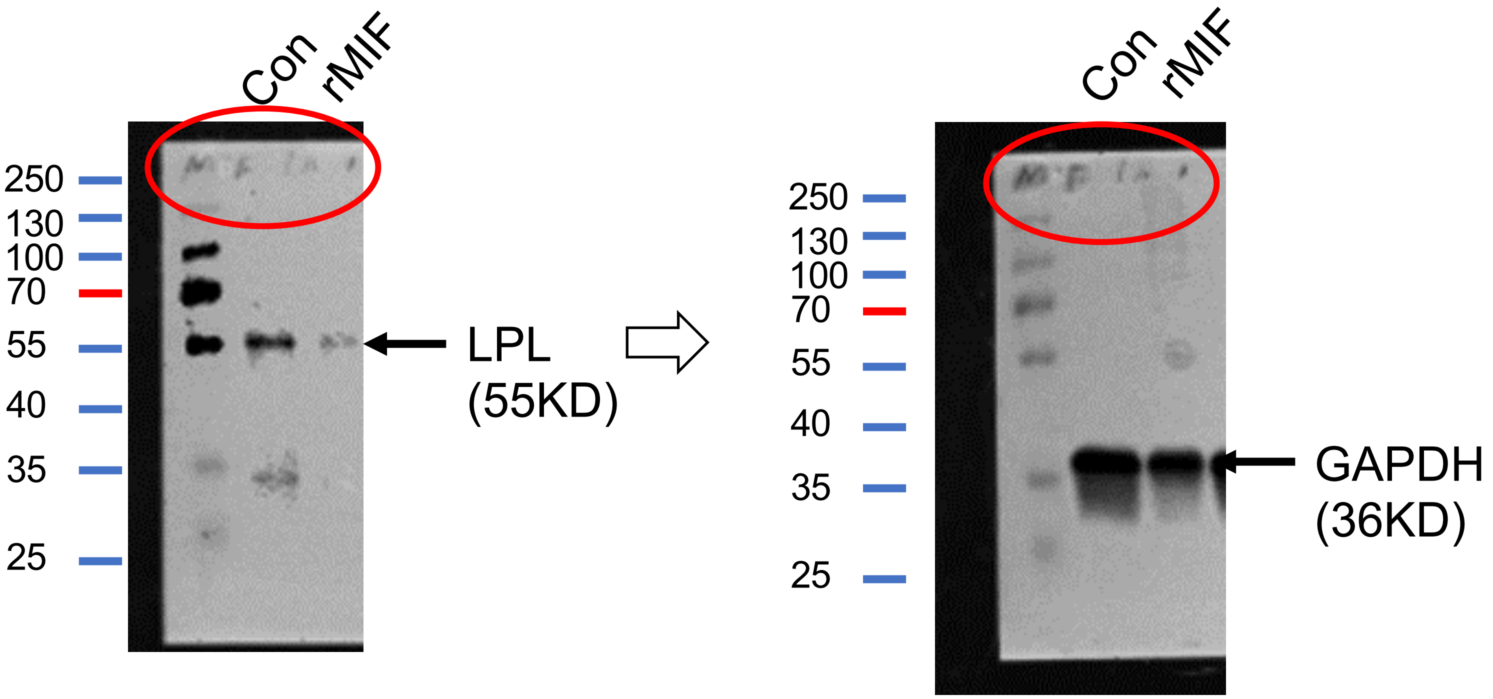

Figure 4F

The PVDF membrane was initially stained with LPL antibody and exposed for 5 mins

The membrane was then stained with GAPDH antibody and exposed for 5 mins

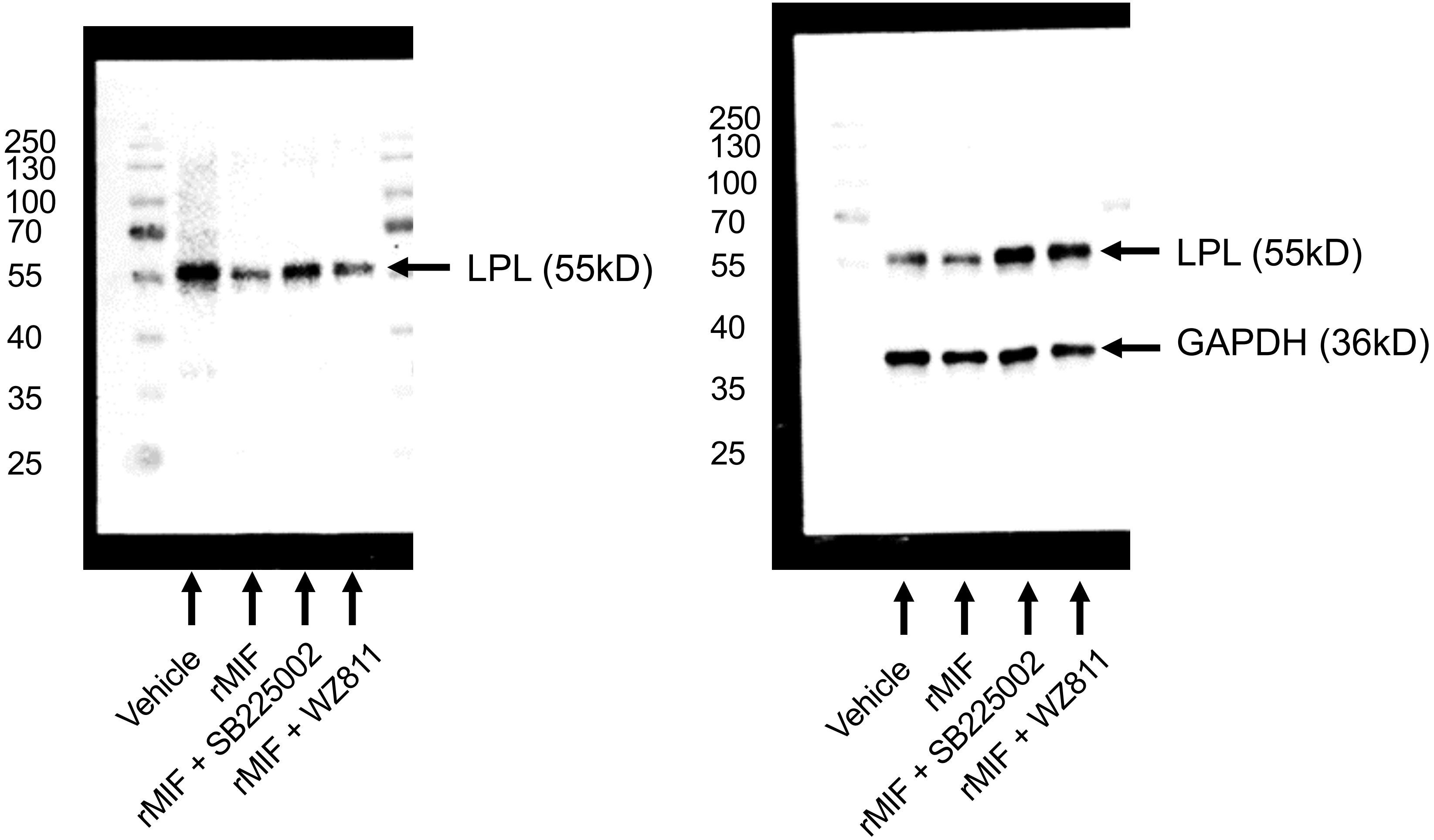

Figure 4F

The PVDF membrane was initially stained with P-Akt antibody and exposed for 1 min

The same membrane was then stained with t-Akt antibody and exposed for 1 min

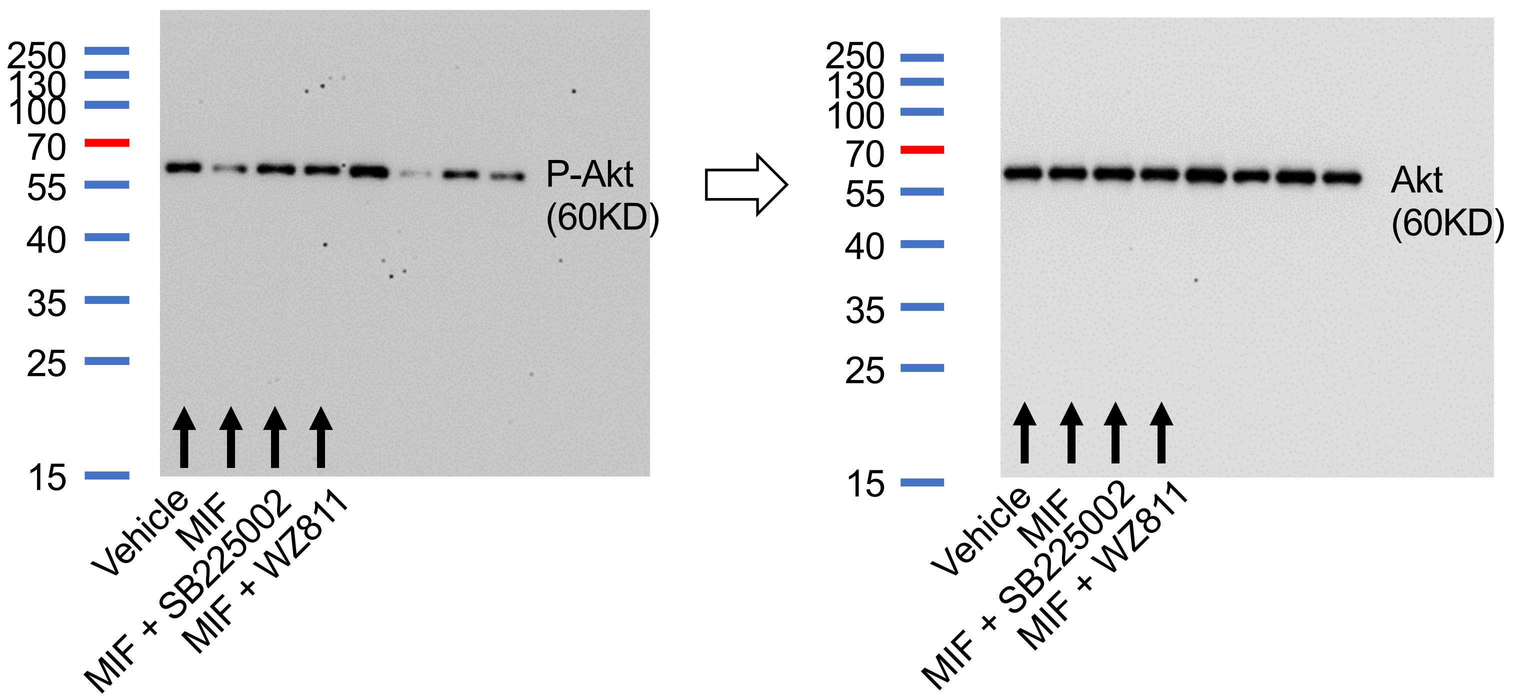

Figure 4G

The PVDF membrane was initially stained with P-Akt antibody and exposed for 1 min

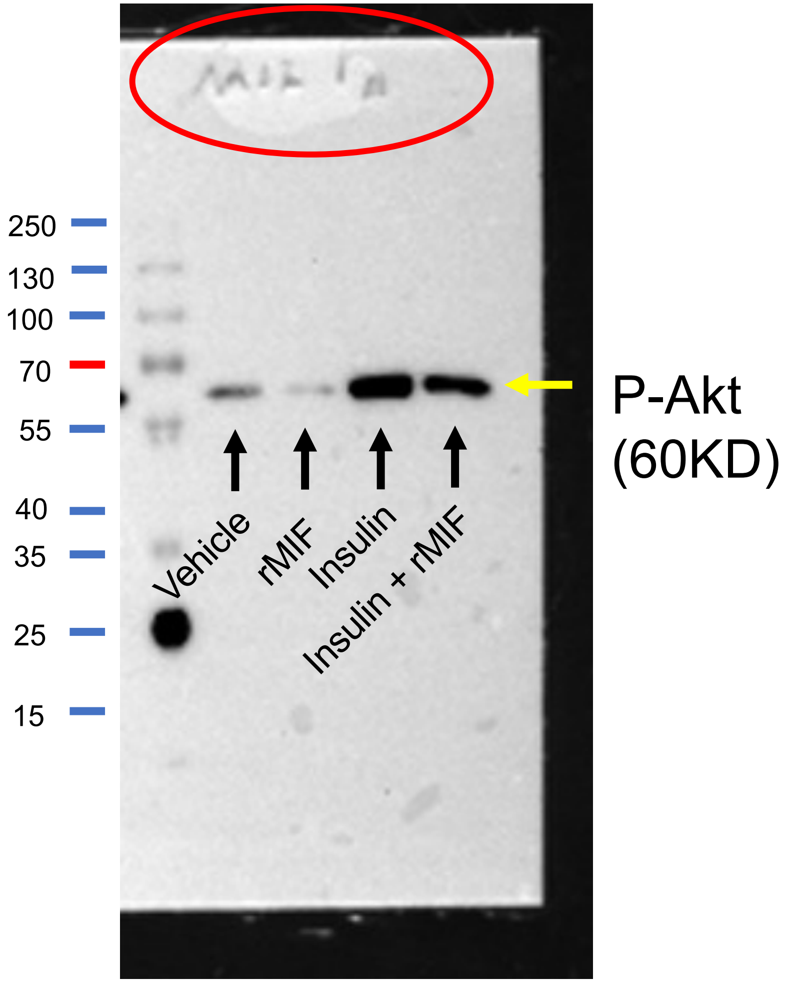

The same membrane was then stained with t-Akt antibody and exposed for 1 min

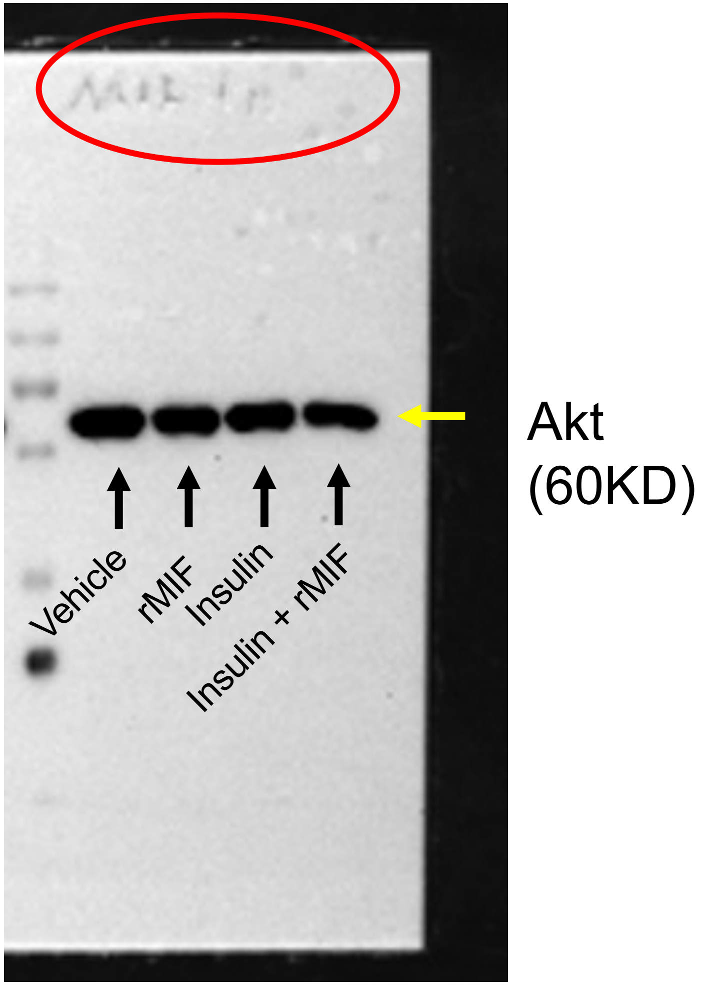

Figure 4G

The PVDF membrane was initially stained with LPL antibody and exposed for 5 mins

The same membrane was subsequently stained with GAPDH antibody and exposed for 5 seconds

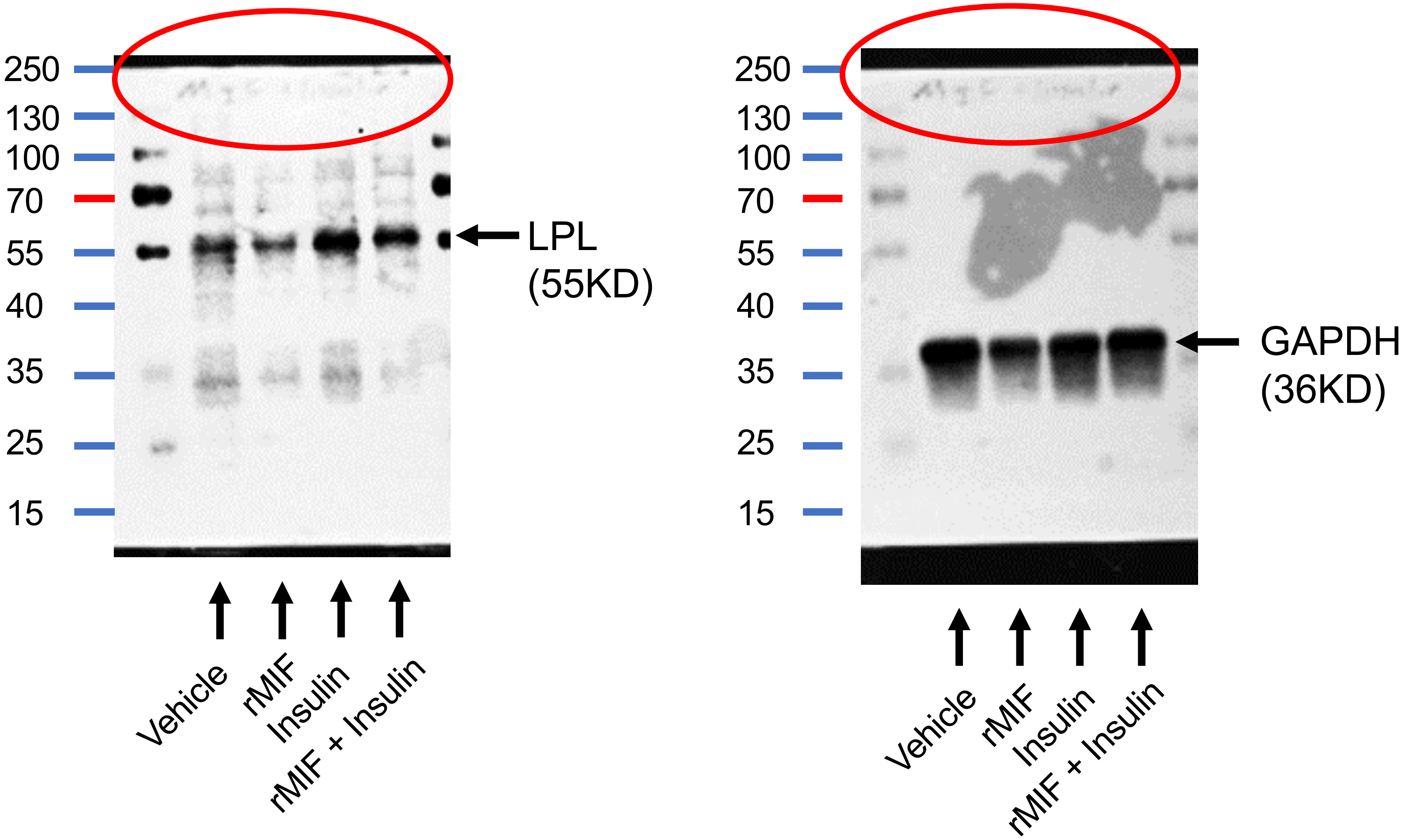

Figure 5B

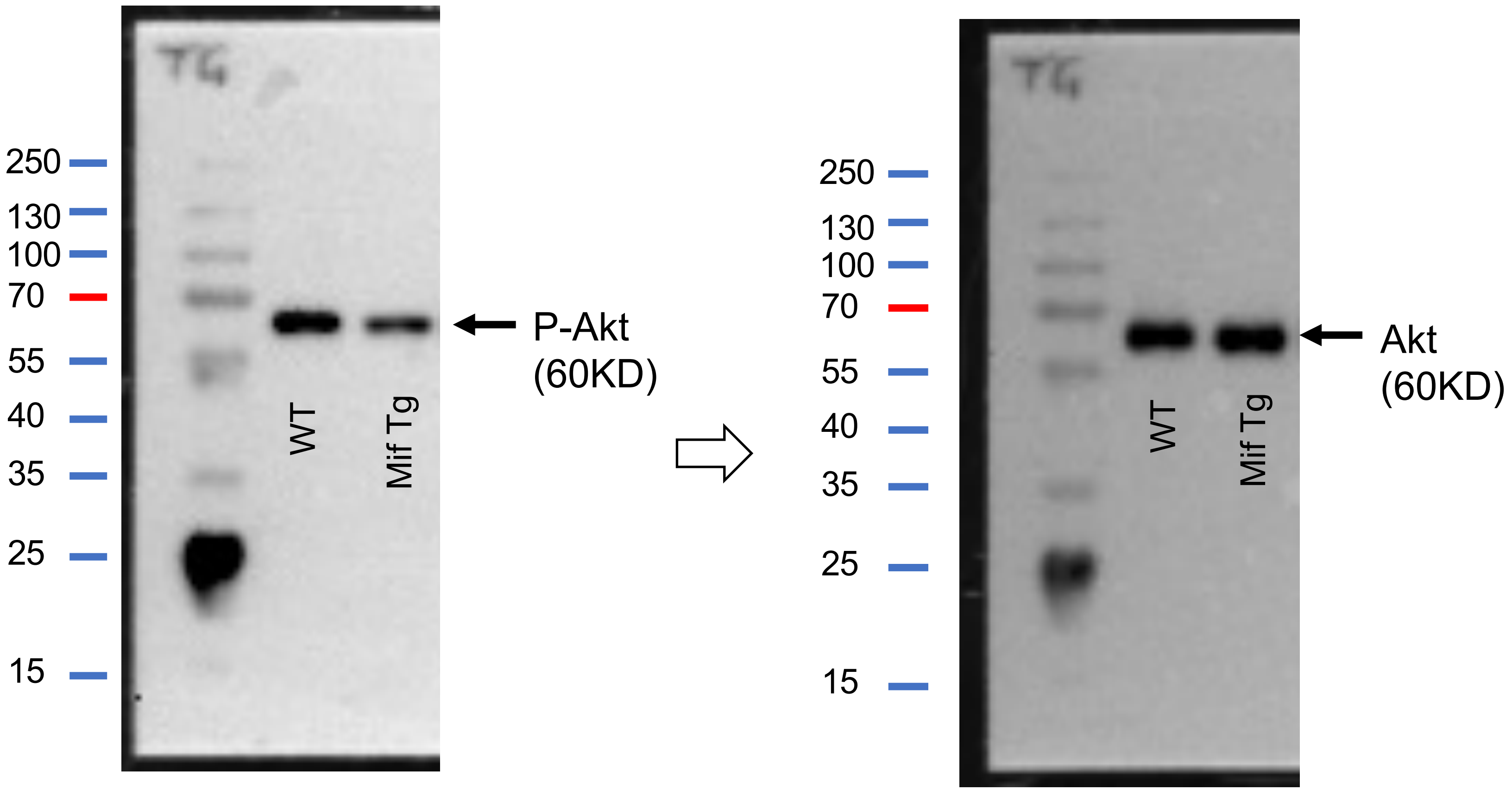
TG
250
130
100
70
55
40
35
25
15
WT
Mif Tg
P-Akt
(60KD)
TG
250
130
100
70
55
40
35
25
15
WT
Mif Tg
Akt
(60KD)

Figure 5D

The PVDF membrane was initially stained with LPL antibody and exposed for 5 mins

The membrane was then stained with GAPDH antibody and exposed for 5 seconds

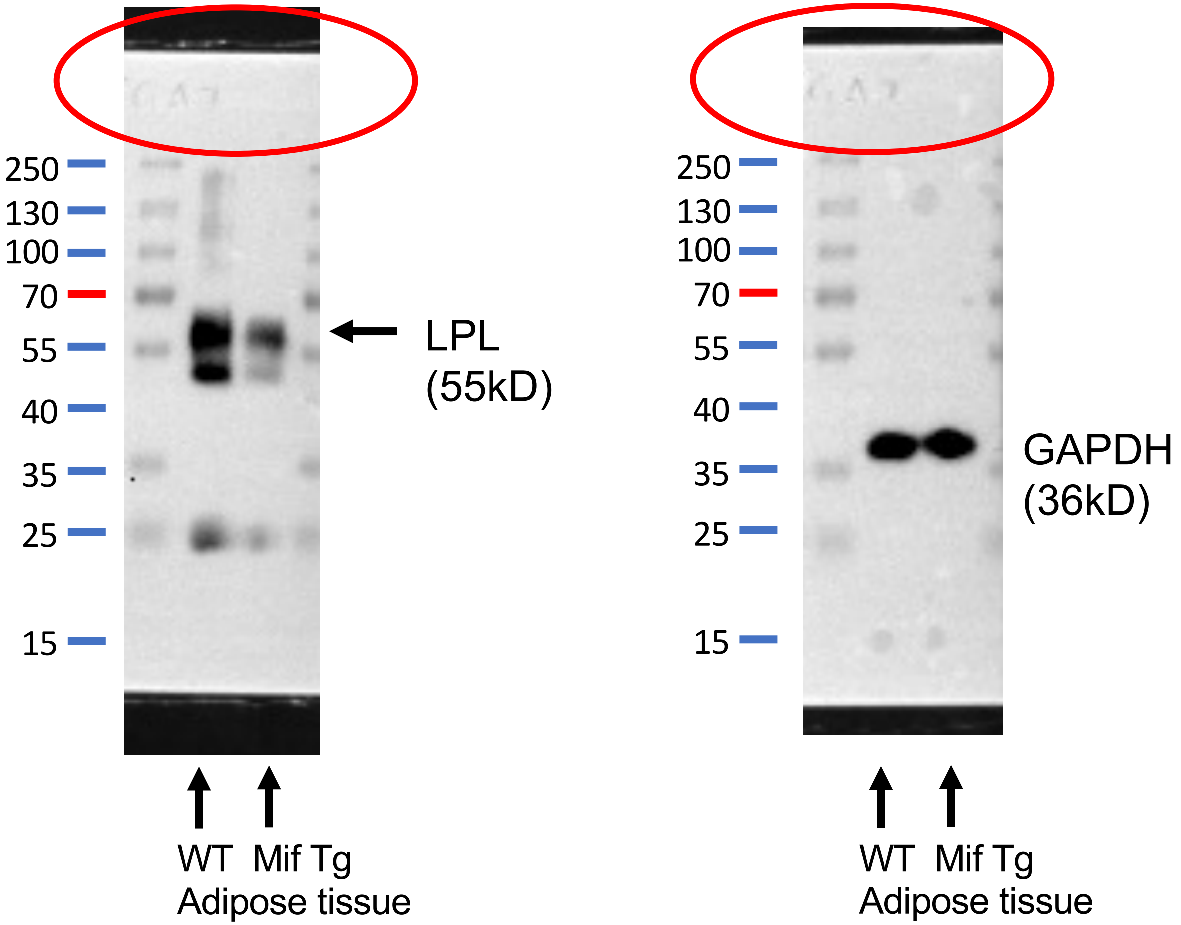

Figure 6C

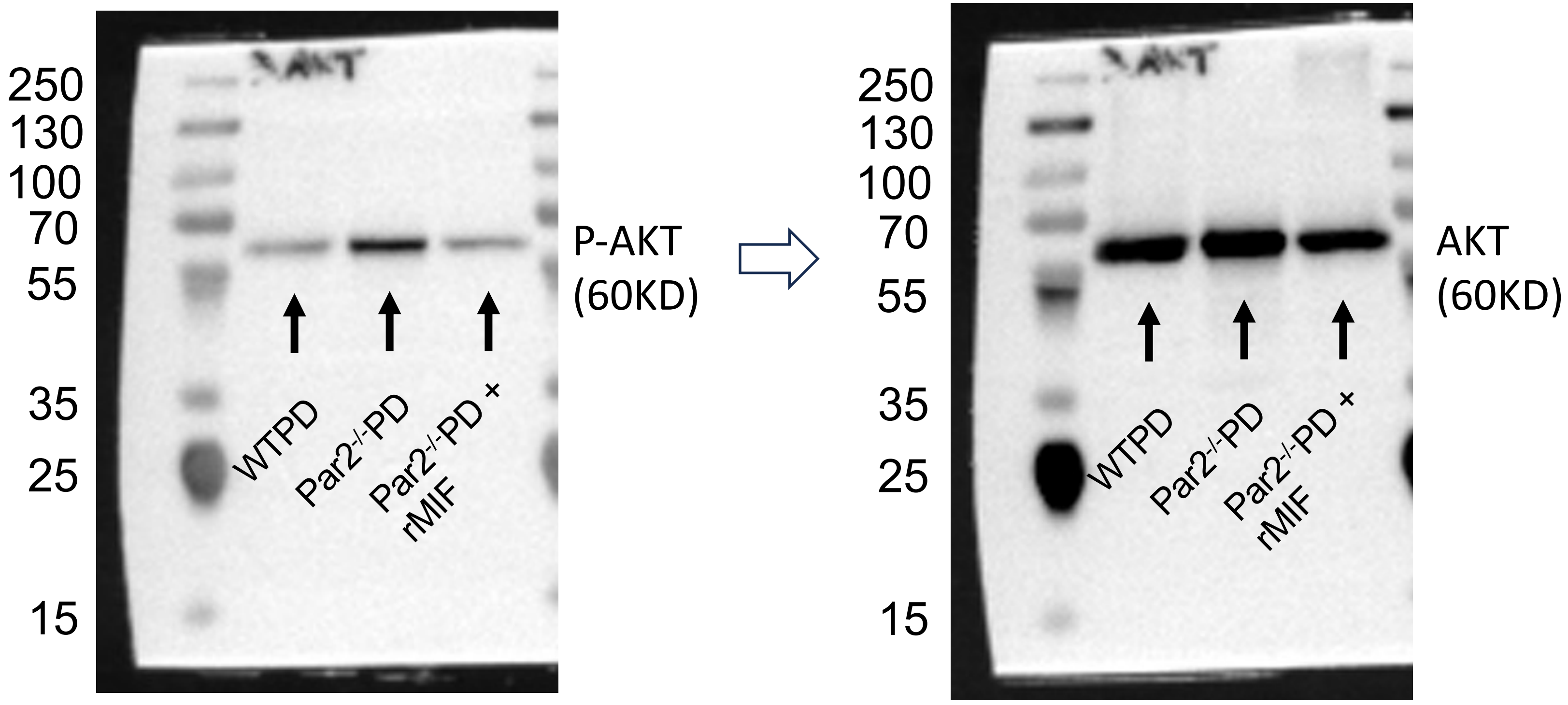
250
130
100
70
55
35
25
15
P-AKT
(60KD)
WTPD
Par2-/-PD
Par2-/-PD +
rMIF
AKT
(60KD)

Figure 6E

The PVDF membrane was initially stained with LPL antibody and exposed for 5 mins

The same membrane was subsequently stained with GAPDH antibody and exposed for 5 seconds

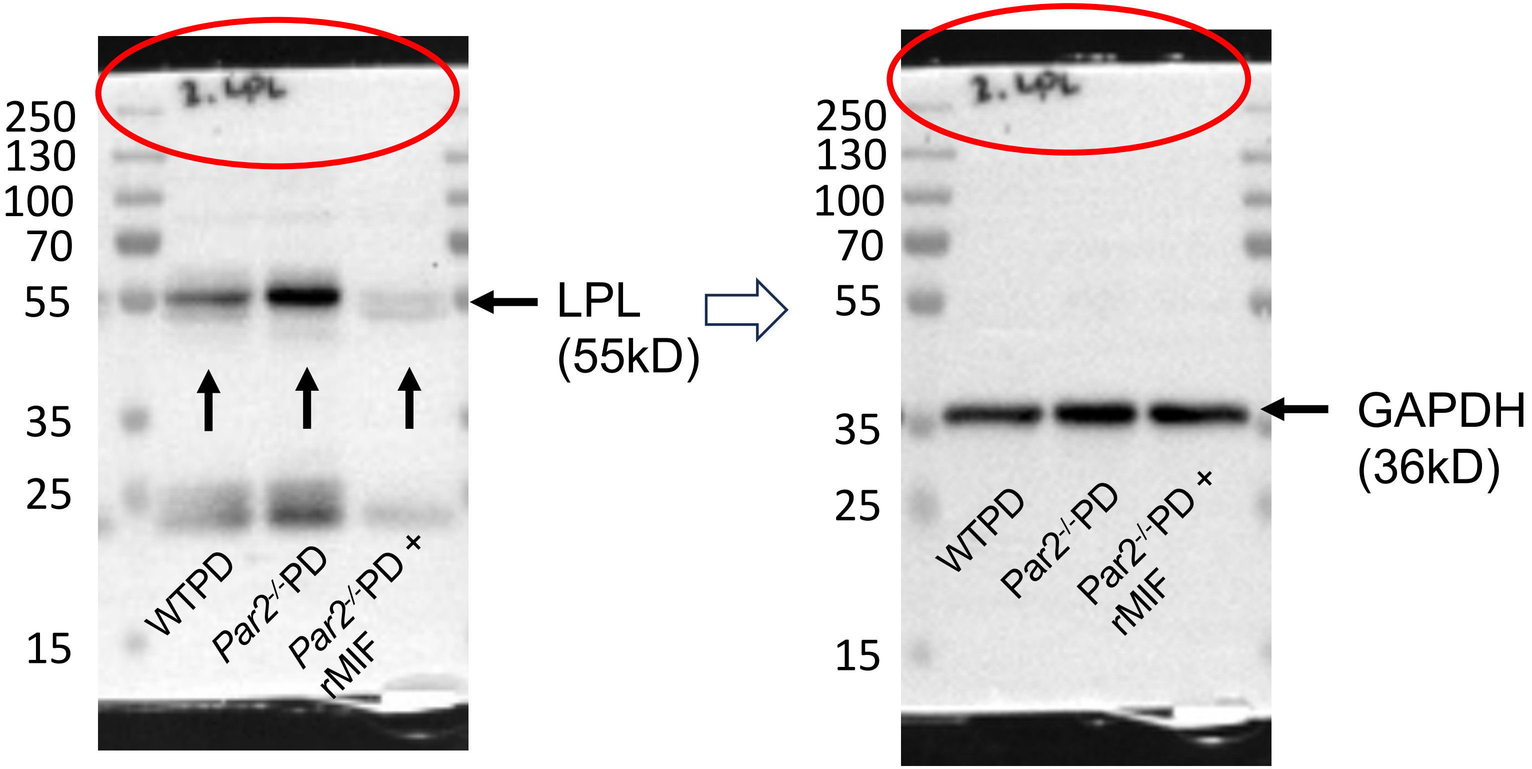

Supplemental Figure 1A

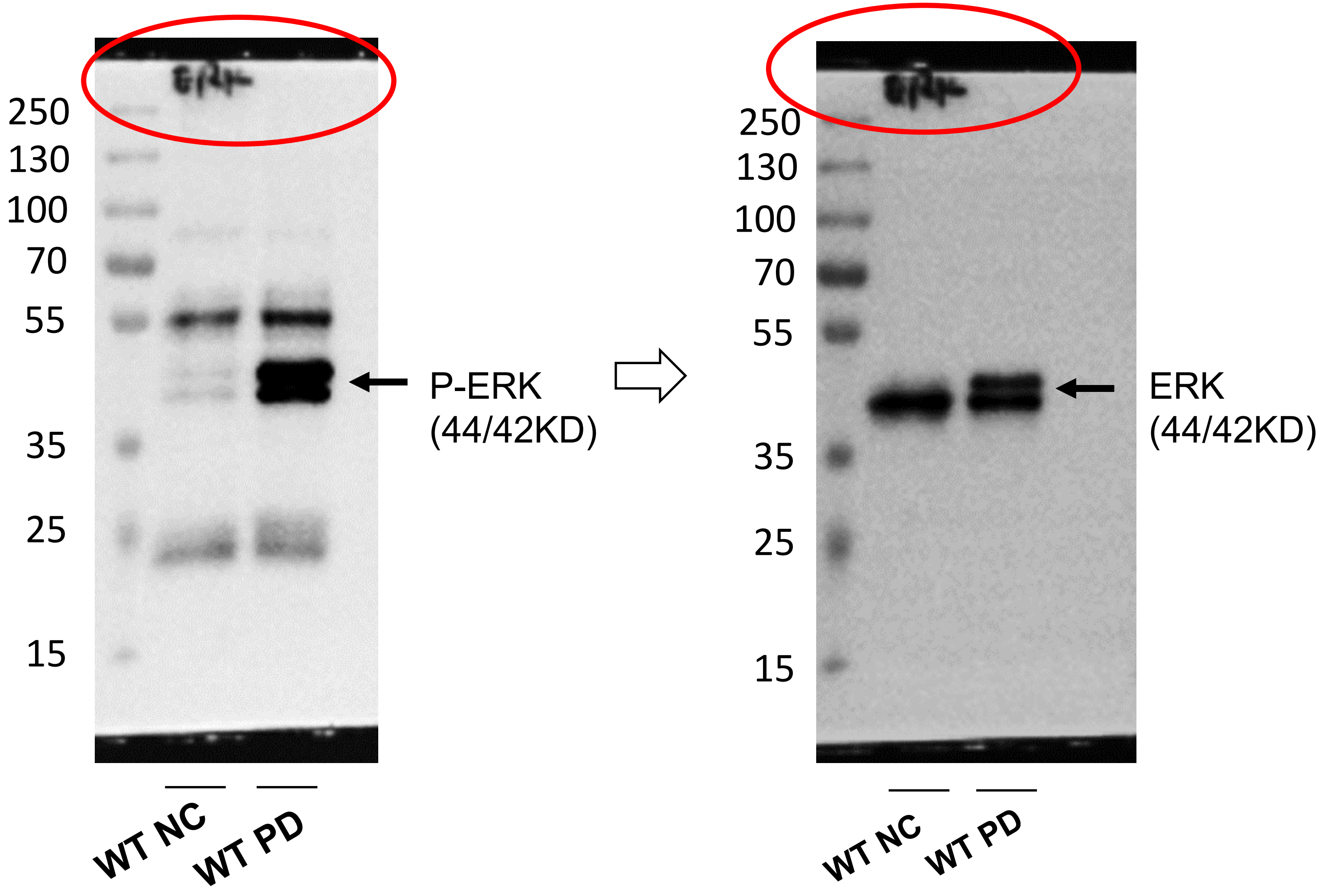

Supplemental Figure 3A

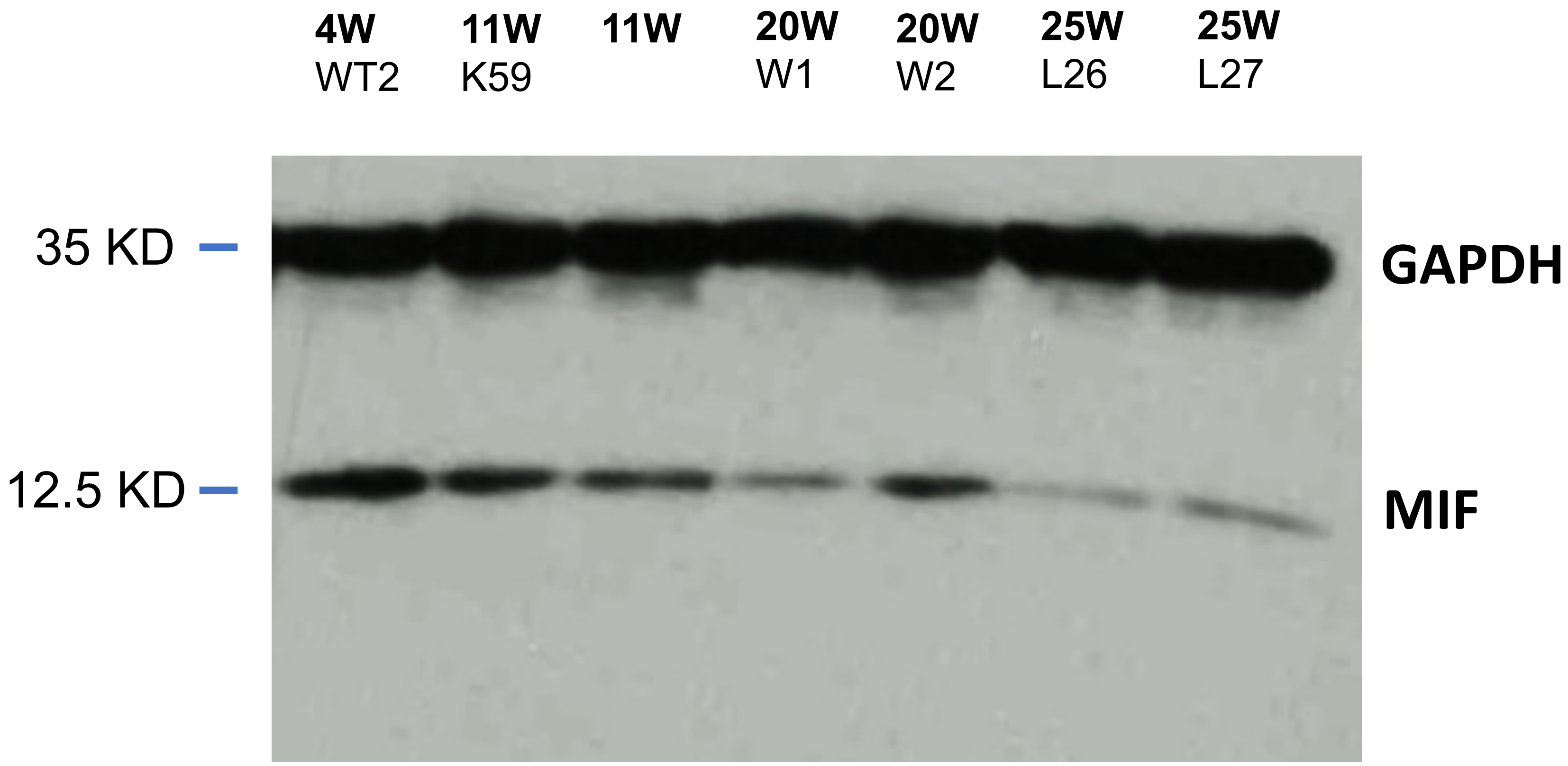

Supplemental Figure 4A

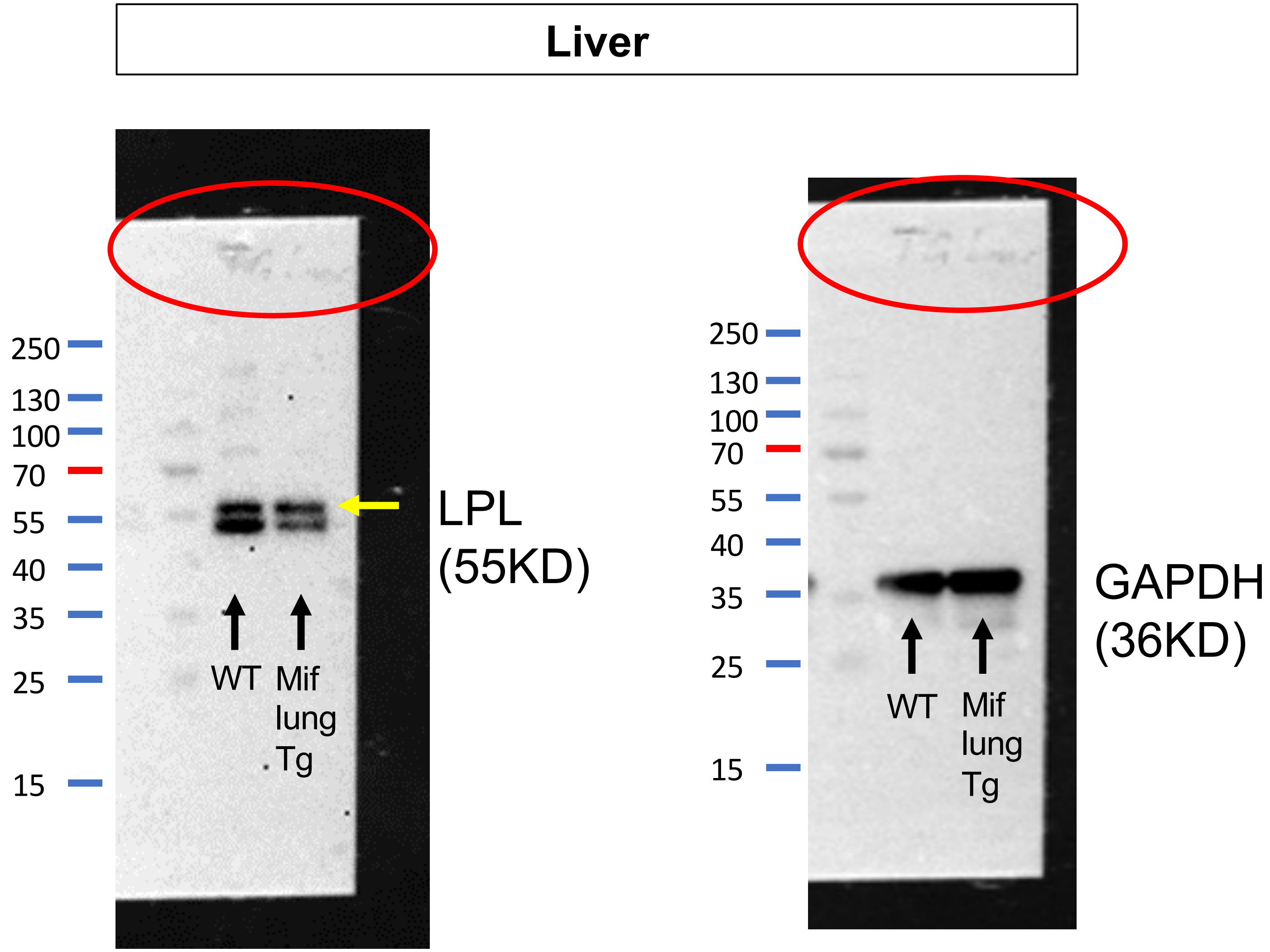

Supplemental Figure 4B

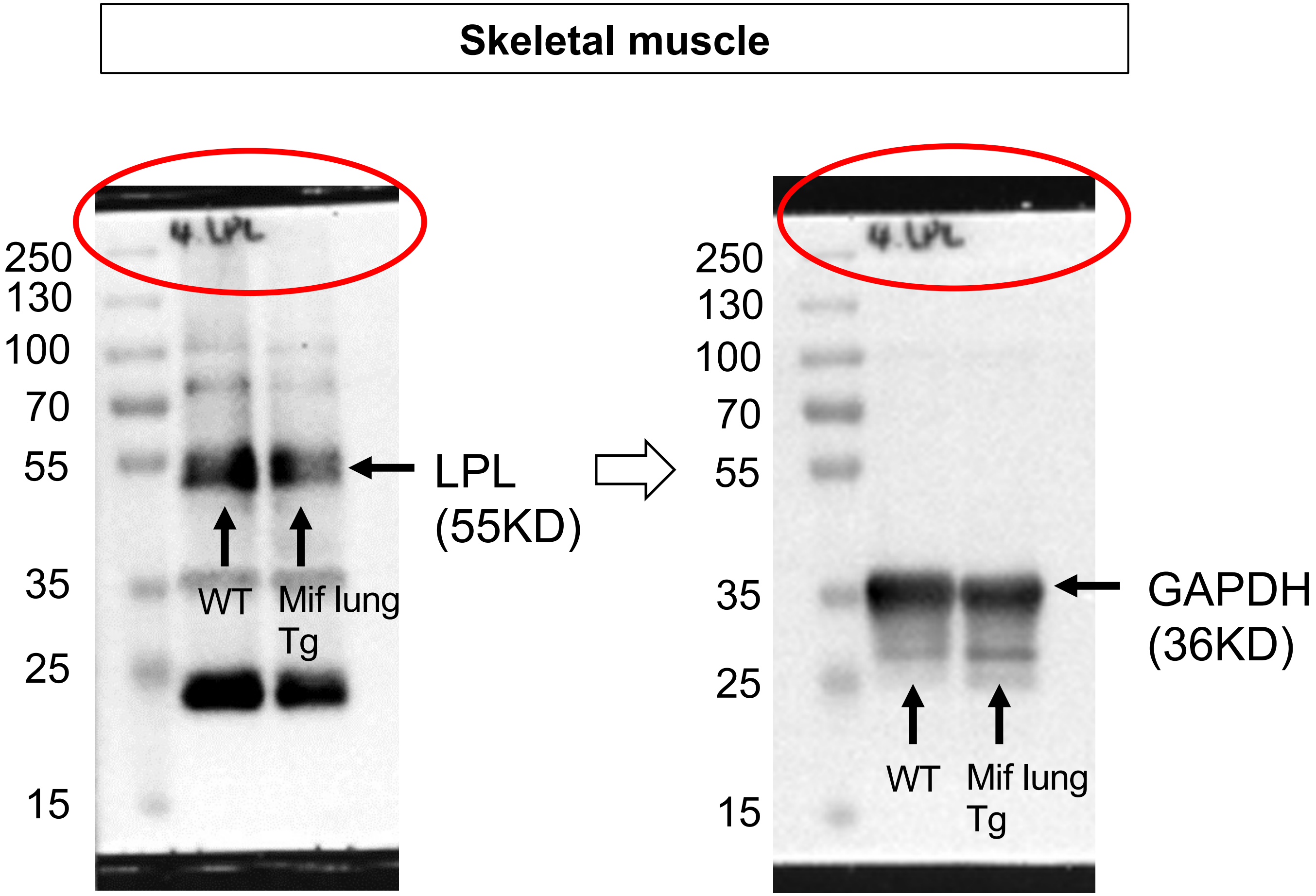

Supplement: Unedited blot and gel images [file jciinsight-9-173240-s048.pdf]
